# Successive modification of polydentate complexes gives access to planar carbon- and nitrogen-based ligands

**DOI:** 10.1038/s41467-019-09367-8

**Published:** 2019-04-02

**Authors:** Xiaoxi Zhou, Xin Pang, Liming Nie, Congqing Zhu, Kaiyue Zhuo, Qingde Zhuo, Zhixin Chen, Gang Liu, Hong Zhang, Zhenyang Lin, Haiping Xia

**Affiliations:** 1 State Key Laboratory of Physical Chemistry of Solid Surfaces and Collaborative Innovation Centre of Chemistry for Energy Materials (iChEM), College of Chemistry and Chemical Engineering, Xiamen University, 361005 Xiamen, China; 2 State Key Laboratory of Molecular Vaccinology and Molecular Diagnostics, Centre for Molecular Imaging and Translational Medicine, School of Public Health, Xiamen University, 361102 Xiamen, China; 3 Department of Chemistry, The Hong Kong University of Science and Technology, Clear Water Bay, Kowloon, HK Hong Kong; 4 Present Address: State Key Laboratory of Coordination Chemistry, Jiangsu Key Laboratory of Advanced Organic Materials, School of Chemistry and Chemical Engineering, Nanjing University, 210023 Nanjing, China

## Abstract

Polydentate complexes containing combinations of nitrogen and carbon (N and C) ligating atoms are among the most fundamental and ubiquitous molecules in coordination chemistry, yet the formation of such complexes with planar high-coordinate N/C sites remains challenging. Herein, we demonstrate an efficient route to access related complexes with tetradentate CCCN and pentadentate CCCCN and NCCCN cores by successive modification of the coordinating atoms in complexes with a CCCC core. Combined experimental and computational studies reveal that the rich reactivity of metal-carbon bonds and the inherent aromaticity of the metallacyclic skeletons play key roles in these transformations. This strategy addresses the paucity of synthetic approaches to mixed N/C planar pentadentate chelating species and provides valuable insights into the synthesis of carbon-based high-coordinate complexes. Furthermore, the resulting complexes are the examples of organometallic species with combined photoacoustic, photothermal, and sonodynamic properties, which makes them promising for application in related areas.

## Abstract (web summary)

Coordination complexes based on polydentate ligands that contain both nitrogen and carbon ligating atoms are ubiquitous, but design of those with planar cores remains challenging. Here the authors show that complexes with planar CCCN, CCCCN and NCCCN cores can be accessed by modification of the coordinating atoms in CCCC core complexes.

## Introduction

**Fig. 1:**
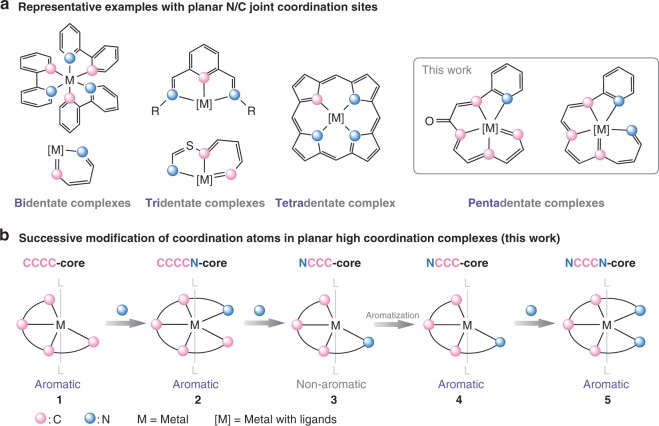
Background and schematic outline of the strategy. **a** Representative examples of polydentate complexes with planar nitrogen/carbon (N/C) joint coordination sites. **b** Successive and direct transformation of coordinating atoms in complexes with a CCCC core via reactions of the metal–carbon bonds to generate planar N/C high-coordinate complexes (this work)

Complexes with polydentate ligands have played a fundamental role in chemistry^1,2^. The most common coordinating atoms in polydentate ligands are donor heteroatoms, such as nitrogen, phosphine, oxygen, and sulfur^3,4^. Notably, carbon can also serve as a coordinating atom in a polydentate complex, and it usually appears in combination with other classical donor atoms^5,6^; such complexes have been theoretically and experimentally examined due to their unique structural, electronic, aromatic, catalytic, and optical properties^7–10^. Among these polydentate complexes, planar systems involving both nitrogen and carbon donors have flourished and have attracted great attention in recent decades^11,12^. As shown in Fig. 1a, complexes with combinations of N and C coordination sites, such as bidentate NC cores^13,14^, tridentate NCN cores^15,16^, and CNC/CCN cores^17,18^, exhibit multiple functions and have had a substantial impact on emerging areas in chemistry. For complexes with higher planar coordination configurations, examples are limited, and the majority of coordinating sites are occupied by nitrogens. Representative examples include the tetradentate complexes known as carbaporphyrinoid systems, in which one or two pyrrolic units of the porphyrin motif are replaced by inverted pyrrolic rings or carbocyclic units (Fig. 1a)^19–22^. To date, the chemistry of molecules with higher planar carbon/nitrogen (C/N) coordination sites, such as those allowing penta- and hexacoordination, remains unexplored owing to the inaccessibility of the atypical geometries, especially for the complexes based on transition metal centers.

As a distinctive family of complexes, metallaaromatics have attracted considerable attentions in recent years^23–29^, and their inherent aromaticity facilitates the bonding of carbon chains with a variety of transition metals. The conjugated carbon chains in these complexes have been viewed as carbon-based polydentate ligands, although these ligands differ from classic ligands in coordination and dissociation. Metallaaromatics with bidentate NC cores (metallapyridines)^14^ or tridentate CCN cores (metallabenzothiazoliums)^18^ have been demonstrated (Fig. 1a). Inspired by the rich reactivities of metal–carbon bonds, we attempted to directly modify metallaaromatic complexes with planar carbon ligands in the hope of generating planar polydentate ligand systems with high N/C combined coordination.

Herein, we report the successive transformation of the coordinating atoms in complex with a CCCC core, which can undergo unique changes in molecular topology, such as a shift of the aromatic unit. Efficient modifications can lead to the discovery of a series of carbon-based high-coordinate N/C ligand systems and the unique planar N/C pentadentate complexes (Fig. 1b), which involve metal centers located at the center of planar four-, five-, or six-membered metallacycles. Interestingly, the resulting complexes exhibit good photoacoustic (PA), photothermal, and sonodynamic properties, making them potentially applicable as theranostic agents.

## Results

### From CCCC to CCCCN complexes

**Fig. 2:**
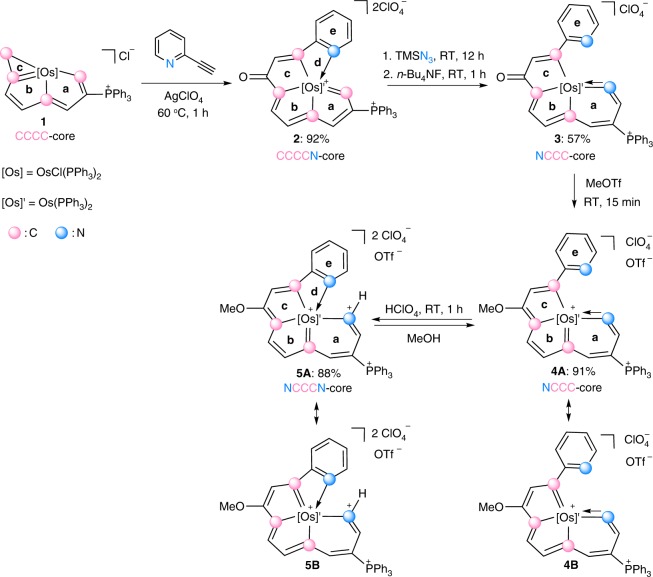
Successive transformations of polydentate complexes. Transformations of CCCC (**1**) to CCCCN (**2**), NCCC (**3**, **4**), and NCCCN (**5**) type complexes

For common, high-coordination polydentate complexes, the ordered equatorial arrangement of the coordinating atoms provides a unique environment for metal binding, hindering the direct replacement of coordinating atoms. However, the strained metallacyclopropene unit in CCCC complex **1** exhibits a strong tendency to undergo ring expansion^30–32^. We thus attempted reactions of **1** with various nitrogen-containing reagents. As shown in Fig. 2, the reaction of complex **1** with 2-ethynylpyridine in the presence of AgClO_4_ generates CCCCN complex **2** in 92% yield. We inferred that AgClO_4_ might serve as a dechlorinating/oxidizing agent to facilitate the [3 + 2] cycloaddition and deprotonation steps. An ^18^O-labeling experiment indicates that the oxygen atom on C8 comes from the trace amount of H_2_O existing in solution. A possible mechanism for the formation of **2** is presented in Supplementary Figure 1.

**Fig. 3:**
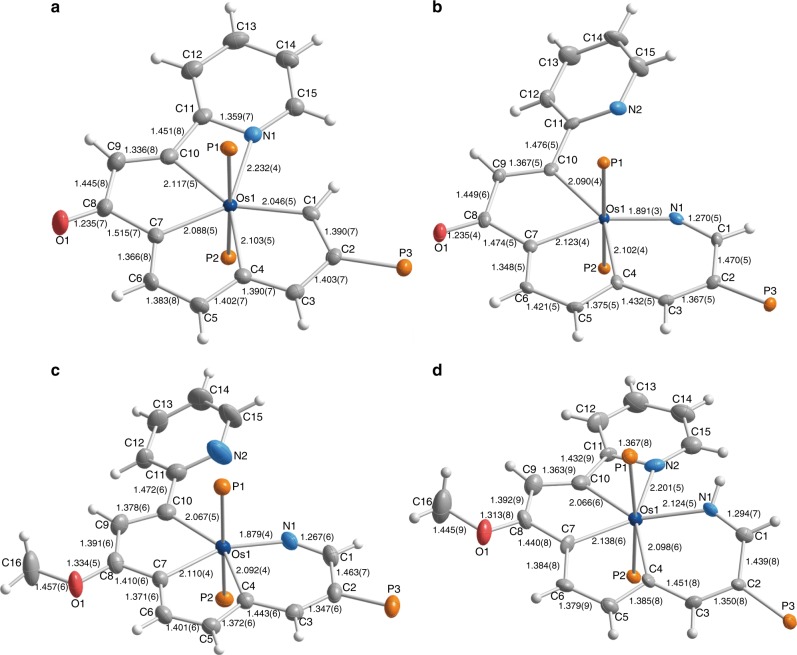
X-ray structures of the cationic parts of **2** (**a**), **3** (**b**), **4** (**c**), and **5** (**d**). Thermal ellipsoids are set at the 50% probability level. The phenyl groups in the PPh_3_ moieties are omitted for clarity

Complex **2** was characterized by spectroscopic and analytical techniques, and its solid-state structure was determined by single-crystal X-ray diffraction (Fig. 3a and Supplementary Figure 2). Interestingly, the CCCC Os coordination in **1** was converted to CCCCN Os coordination in **2**. The metal center in **2** features a pentagonal bipyramidal geometry, and the five coordination positions in the equatorial plane are occupied by four carbon atoms and one nitrogen atom. The equatorial polycyclic ring system (rings **a**, **b**, **c**, **d**, and **e**) consists of 17 atoms (Os1, N1, and C1–C15), which are nearly coplanar, as reflected by the small mean deviation from the least-squares plane (0.049 Å). The bond distances of rings **a** and **b** are within the range of typical electron-delocalized systems and similar to those observed in osmapentalenes^33^, whereas the bonds in rings **c** and **d** display obvious deviations from their ideal distances. The Os1−N1 bond length is 2.232(4) Å, which is comparable to lengths typically reported for coordinated pyridine units (bond lengths and angle ranges in this article are all based on a search of the Cambridge Structural Database, CSD version 5.39 in November 2017), indicating its dative bond character. Complex **2** exhibited a nuclear magnetic resonance (NMR) spectrum consistent with the putative fused metallapentalene, and the ^1^H chemical shifts of rings **a** and **b** (δ 14.2 (C^1^*H*), 9.1 (C^3^*H*), 9.7 (C^5^*H*), and 8.1 (C^6^*H*) parts per million (ppm)) are similar to those reported for metallapentalene species^33^.

### From CCCCN to NCCC complexes

The bond length of Os1−C1 in **2** is 2.046(5) Å, and the chemical shifts of this unit are 14.2 ppm for C1*H* and 232.4 ppm for C1, indicating its carbene character. Therefore, we investigated reactions of **2** with nucleophiles. Indeed, treating **2** with azidotrimethylsilane (TMSN_3_) and tetrabutylammonium fluoride (*n*-Bu_4_NF) led to the formation of complex **3** with a tetradentate ligand (Fig. 2).

The solid-state structure of **3** was probed by single-crystal X-ray diffraction (Fig. 3b and Supplementary Figure 3), which confirmed the formation of a ring expansion product from the parent five-membered ring. The tricyclic topology involves a six-membered aza-metallacycle fused to two five-membered metallacycles, and the equatorial coordination sites around the osmium center are filled by the NCCC-type tetradentate ligand. Notably, the inserted nitrogen atom is incorporated in cumulated double bonds, as indicated by the Os1−N1 bond length of 1.891(3) Å and the N1−C1 bond length of 1.270(5) Å. The Os1−N1−C1 bond angle of the cumulated double bonds deviates considerably from linearity (144.6(3)°), and the angle is smaller than all previously reported bond angles of azavinylidene complexes (150.9°–179.6°). Complex **3** contains a considerable alternation in the carbon−carbon bond lengths in rings **a** and **b**, indicating a change in the aromaticity of the metallacycles compared to those in complex **2**. As with the results of X-ray diffraction, the ^1^H NMR signals of rings **a** and **b** in **3** are distinct from those observed in **2**; the protons of the metallacycles exhibited obvious up-field shifts to δ 4.5(C^1^*H*), 5.9 (C^3^*H*), 5.4 (C^5^*H*), and 7.5 (C^6^*H*) ppm. The up-field shifts of the resonances are likely due to the dearomatization of the metallapentalene unit via the nitrogen insertion reaction.

**Fig. 4:**
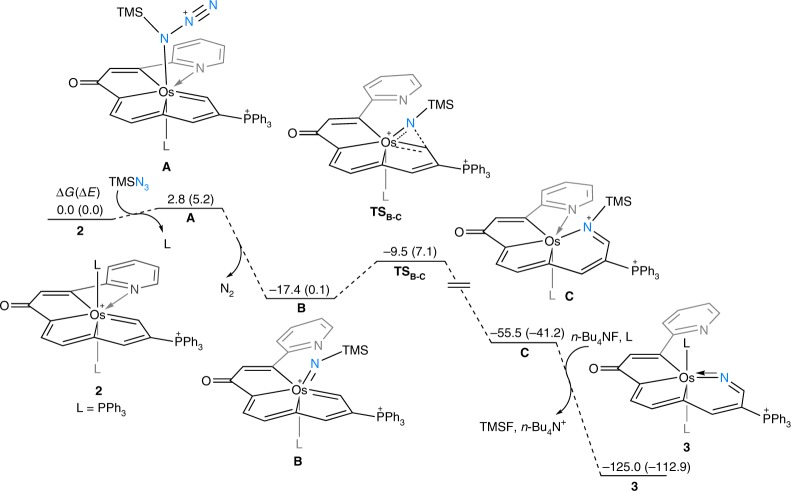
Calculated energy profile for the formation of structure **3**. The relative Gibbs free energies and electronic energies (within parentheses) are given in kcal mol^−1^

Of particular note in the transformation of seven-coordinate **2** to six-coordinate **3** is the change in the C/N incorporated polydentate ligands. The observation that treatment of a complex containing a pentadentate CCCCN core with an azide yields a product having the NCCC core was a great surprise owing to the known stability and inertness of pentadentate chelates, and in the literature, they mainly undergo typical ligand substitution or dissociation reactions^32,34^. Figure 4 shows the calculated energy profile based on the proposed mechanism for the experimentally observed transformation of **2** to **3**. The ligand substitution of PPh_3_ with azidotrimethylsilane is followed by the release of N_2_, which initiates the reaction to generate the intermediate **B**. The nitrogen insertion step, which involves a structural rearrangement to give the six-membered intermediate **C**, is kinetically favorable with a small barrier of 7.9 kcal mol^−1^. In addition, the intermediate **C** is significantly more stable than the intermediate **B**, as a result of the C−N bond formation. Finally, elimination of the trimethylsilyl group in the presence *n*-Bu_4_NF and re-coordination of a PPh_3_ ligand produce the final product **3**.

### The methylation of the NCCC complex

To aromatize **3**, we chose methyl trifluoromethanesulfonate (MeOTf) as the electrophile. As shown in Fig. 2, reaction of **3** with MeOTf afforded complex **4**, in which the original carbonyl group was methylated, in high yield. The ^1^H NMR spectrum of **4** clearly shows the protons of the metallacycles at δ 5.6 (C^1^*H*), 7.0 (C^3^*H*), 7.0 (C^5^*H*), 8.8 (C^6^*H*), and 8.3 (C^9^*H*)ppm, which are considerably lower field than those of complex **3** (4.5 (C^1^*H*), 5.9 (C^3^*H*), 5.4 (C^5^*H*), 7.5 (C^6^*H*), and 7.4 (C^9^*H*)ppm), suggesting that the tricyclic framework was aromatized. The aromatic structure indicated by the NMR spectra was further confirmed by a single-crystal X-ray diffraction study of **4** (Fig. 3c and Supplementary Figure 4). Visually, the structure appears similar to complex **3**, as the metal center is also six-coordinate and bound to an NCCC-type tetradentate ligand. The most prominent difference is that complex **4** has a fused metallapentalenoid configuration in which all the bond distances in the metallacycles clearly fall in the aromatic range. The mean deviation from the least-squares plane through Os1, N1, and C1–C10 is 0.032 Å, indicating good planarity of the tricyclic ring system. The bond lengths of the Os−C and C−C bonds within the two five-membered metallacycles are comparable to those reported for metallapentalenes (1.926−2.139 Å for the Os−C bond lengths and 1.367−1.410 Å for the C−C bond lengths)^33^, which is consistent with the assignment of a metallapentalene unit. Together with the fused six-membered aza-metallacycle (ring **a**), the core of complex **4** can be described as a metal-containing pentalene system fused to a pyridine. The structural parameters suggest contributions from both resonance forms, **4A** and **4B** shown in Fig. 2. Remarkably, complex **4** represents a unique aromatic scaffold in which the metal center is shared by two five-membered rings and a six-membered ring. Considerable progress has been made in the synthesis of metallaaromatics with various scaffolds^23–29^. However, synthetic route to metallapyridines is very limited^14,35,36^. Complex **4** represents a rare example of fused-ring metallapyridine framework. In 2012, we reported a sulfur-tethered metallapyridine complex through an annulation reaction of a metallabenzene derivative^35^. The metallapyridine unit of the complex can be viewed as *m*-metallapyridine, in which the nitrogen atom is not adjacent to the metal center. By comparison, the *o*-metallapyridine unit in complex **4** is similar to the previous monocyclic examples tantalapyridine^36^ and osmapyridine^14^.

### Conversion of NCCC complexes to NCCCN complexes

The addition of excess acid to NCCC-type complex **4** in solution resulted in the re-coordination of the pyridine unit to the metal center. As shown in Fig. 2, the protonation of the nitrogen atom in the osmapyridine unit (ring **a**) of **4** in the presence of perchloric acid afforded NCCCN-type complex **5** in 88% isolated yield. These results demonstrate that the nitrogen atom of the osmapyridine unit (ring **a**) in **4** is more reactive towards electrophiles than that of the pyridine (ring **e**), which can be rationalized by discrete fourier transform (DFT) calculations (Supplementary Figure 6). The calculated highest occupied molecular orbital (HOMO) of **4** is mainly located on the metallacycles. Interestingly, the protonation of **4** is reversible. Treatment of **5** with a protic solvent, such as methanol, leads to the elimination of the proton and quantitatively regenerates **4**.

The identification of **5** was supported by solution and solid-state characterization techniques. The solid-state structure confirmed the presence of a seven-coordinate osmium center, in which two of the coordination sites are similar to those in **2** (two axial phosphine ligands), and the other five coordination sites in the equatorial plane are occupied by three carbon atoms and two nitrogen atoms (Fig. 2d and Supplementary Figure 5). The bond distances within the two five-membered metallacycles and the six-membered metallacycle vary slightly from those in parent compound **4**, whereas the Os1−N1 bond length is markedly elongated to 2.124(5) Å due to the protonation of the nitrogen atom. The structural parameters indicate **5** can be represented by the resonance structures **5A** and **5B**, as a metallapentalene fused metallapyridinium. A closely related monocyclic osmapyridinium has been reported through the formal [4 + 2] cycloaddition reaction of 1-metalla-1,3-dienes with nitriles^14^. The bond lengths of Os1−C4 (2.098(6) Å) and Os1−N1 (2.124(5) Å) in **5** are longer than those of the monocyclic osmapyridinium (Os−C 1.943(9) Å and Os−N 1.952(7) Å)^14^, probably due to the lessened electron delocalization in the fused osmapyridinium ring and the structural distortion induced by steric hindrance (*vide infra*), respectively. The C1−N1 bond length (1.294(7) Å) is almost identical with that of the monocyclic osmapyridinium (1.294(12) Å)^14^, but is longer than that of **4** (1.267(6) Å). Compared to the monocyclic osmapyridine^14^, the osmapyridine units in both complexes **4** and **5** show slight bond distance alternation, which may be caused by the weakened electron delocalization in the fused-ring systems. The bicyclic metallapentalene rings (rings **b** and **c**) and the metallapyridium ring (ring **a**) deviate slightly from planarity, as reflected by their mean deviations from the least-squares plane (0.084 and 0.113 Å, respectively). The overall pentacyclic core of **5** (Os1, N1, N2, C1–C15) displays a noticeable distortion with a mean deviation from the least-squares plane of 0.254 Å, which is probably due to the steric hindrance between the two hydrogen atoms (N1*H* and C15*H*). The involvement of N1*H* in resonance was confirmed by the broad peak at 11.3 ppm in its ^1^H NMR spectrum. The remaining protons on the metallacycle were observed at shifts typical of metallaaromatic protons (9.3 (C^1^*H*), 7.6 (C^3^*H*), 8.3 (C^5^*H*), 8.4 (C^6^*H*), and 8.8 (C^9^*H*)ppm) and are entirely consistent with the delocalized bonding in the three fused metallacycles.

### DFT computational study

To elucidate the bonding and electronic structures of these unique polydentate complexes, we carried out DFT calculations on the simplified unsubstituted models **2′**–**5′** by replacing PPh_3_ with PH_3_. The optimized structural parameters of **2′**–**5′** agree well with those observed in the crystal structures of **2**–**5**, respectively. The trend in the calculated Wiberg bond indices for the Os–C and Os–N bonds of the model complexes **2′**–**5′** is consistent with that in the relevant bond lengths of the X-ray molecular structures (Supplementary Figure 7). Taking the NCCCN-type complex **5′** as an example, the Wiberg bond indices are 0.88, 0.76, and 0.89 for Os1–C4, Os1–C7, and Os1–C10, respectively (Supplementary Figure 7d), suggesting strongly covalent Os–C bonding between the osmium center and the carbons. The Wiberg bond index of Os1–N1 (0.64) is slightly larger than Os1–N2 (0.51), which could be attributed to the π-electron delocalization within the metallapyridinium ring. The main characteristic of the orbital interactions in the aromatic metallacycles of **2′**, **4′**, and **5′** is the involvement of two filled metal *d* orbitals (*d*_*xz*_ and *d*_*yz*_) in the π bonding. The key occupied π molecular orbitals (π-MOs) of complexes **2′**, **4′**, and **5′**, which reflect the π-delocalization along the perimeter of the polycyclic system, are shown in Supplementary Figure 8–10. The MOs are derived principally from the orbital interactions between the *p*_π_ orbitals of the organic fragment and the *d* orbitals of the Os atom (5*d*_*xz*_ and 5*d*_*yz*_). For example, the HOMO and HOMO-13 (π symmetry) in **2′** mainly reflect interactions between the metal *d*_xz_ orbital and the *p*_π_ orbitals of organic fragment orbital, and HOMO-4 and HOMO-9 (δ symmetry) of **2′** show interactions between the metal *d*_yz_ orbital and the *p*_π_ orbitals of organic fragment orbital (Supplementary Figure 8).

**Fig. 5:**
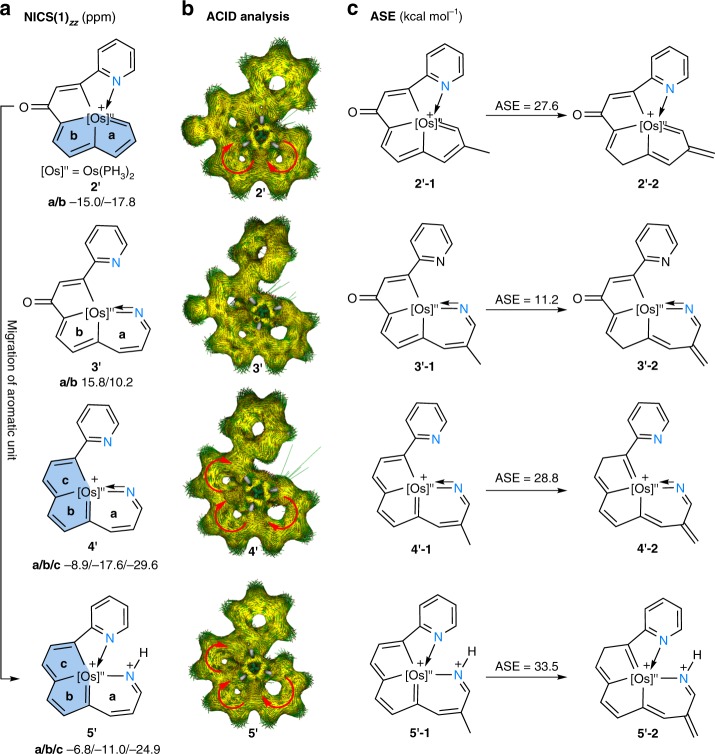
Aromaticity evaluations. **a** NICS(1)_*zz*_ (nucleus-independent chemical shift along the *z*-axis at 1 Å above the ring critical point, ppm) evaluations of the aromaticity of model complexes **2′**–**5′**. Blue rings: The migration of the osmapentalene unit from rings (**a**, **b**) to rings (**b**, **c**). **b** Anisotropy of the current-induced density (ACID) plot of model complexes **2′**–**5′** with an isosurface value of 0.025. The magnetic field vector is orthogonal to the ring plane and points upward. **c** Aromatic stabilization energy (ASE, kcal mol^−1^) evaluations of model complexes **2′**–**5′**

The equalized bond lengths and good stability of the complexes (**2**, **4**, and **5**) and the experimentally observed dearomatization–aromatization process (from **2** to **3** and from **3** to **4**) prompt us to examine the aromaticity of these polycyclic species. DFT computational studies were then carried out to elucidate the aromaticity of the tetra- and pentadentate complexes. We calculated the nucleus-independent chemical shift (NICS) values^37–39^ along the *z*-axis at 1 Å above the ring critical point (NICS(1)_*zz*_) of model complexes (**2′**, **3′**, **4′**, and **5′**). The average value was used when the environments above and below the ring centers were not equivalent. In general, negative NICS values indicate aromaticity. As depicted in Fig. 5a, all the calculated NICS values for complex **2** (rings **a** and **b**), complex **4** (rings **a**, **b**, and **c**), and complex **5** (rings **a**, **b**, and **c**) are negative, which is in sharp contrast with those of model complex **3′** (rings **a** and **b**). The aromaticity of the model complexes is further supported by the anisotropy of the current-induced density (ACID)^40,41^ calculations. As shown in Fig. 5b and Supplementary Figure 11–14, the obvious diatropic ring currents (clockwise vectors) demonstrate the aromaticity of the fused five-membered rings of **2′** and the two fused five-membered rings and six-membered rings of **4′** and **5′**, whereas no clear diatropic or paratropic ring currents could be found in the five-membered ring or the six-membered ring of **3′**.

We also evaluated the aromatic stabilization energy (ASE) by employing the isomerisation method introduced by Schleyer and Pühlhofer^42,43^. As shown in Fig. 5c, ASE values of 27.6, 28.8, and 33.5 kcal mol^*−*1^ were calculated for complexes **2′**, **4′**, and **5′**, respectively; these values are comparable to those of other reported fused metallaaromatics^44^, but are much larger than the calculated ASE value (11.2 kcal mol^*−*1^) of **3′**. Different isomeric model complexes have been tested for the ASE calculations (Supplementary Figure 15), which present similar values. In combination with experimental data, the NICS values and the ACID evaluation, complex **3** is considered as non-aromatic, although the model complex **3′–1** gives positive ASE value. A positive ASE value of non-aromatic **3′–1** may be ascribed to the partial conjugated fragment within the two fused rings (**a** and **b**), which could increase the stability with comparison to the non-conjugated isomer **3′–2**.

These calculation results nicely confirm the aromatic nature of the chelates, which should account for the successive transformation process from the complex containing the CCCCN-type ligand (**2**) to a species containing an NCCCN-type ligand (**5**). More importantly, the dearomatization–aromatization process from **2** to **3** and from **3** to **4** leads to the migration of the metallaaromatic conformation. As shown in Fig. 5a, the insertion of an additional coordinating nitrogen atom results in the original metallapentalene unit in **2** (rings **a** and **b**) rearranging to different positions in the metallacyclic systems in **4** and **5** (rings **b** and **c**). Such an extraordinary shift in the aromatic units has never been observed in metallacyclic species.

### Properties of chelates with N/C coordination environments

**Fig. 6:**
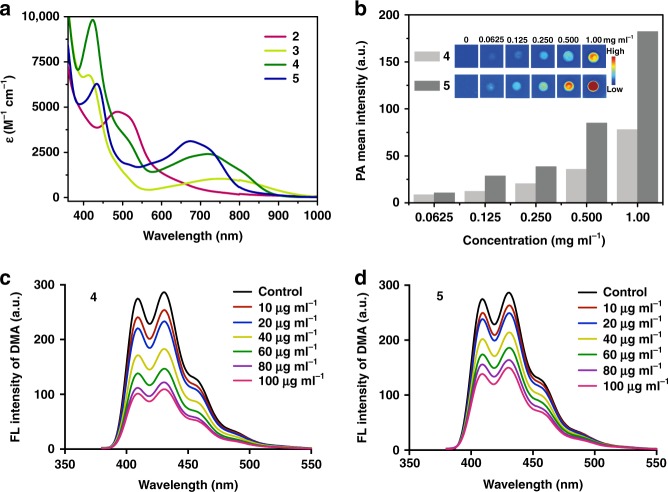
Properties of chelates with N/C coordination environments. **a** Ultraviolet–visible (UV–vis) absorption spectra of complexes **2**–**5** measured in CH_2_Cl_2_ at room temperature. **b** Photoacoustic (PA) mean intensities of **4** and **5** at different concentrations (0.0625, 0.125, 0.250, 0.500, and 1.00 mg ml^−1^) in MeCN monitored at *λ* = 700 nm (Inset: PA imaging). **c**, **d** Assessment of the generation of reactive oxygen species (ROS) by **4** and **5** at different concentrations (10, 20, 40, 60, 80, and 100 μg ml^−1^ in CH_2_Cl_2_) when activated by ultrasound (US) (1 MHz, 0.56 W cm^−2^, 50% cycle, 5 min). FL: fluorescence; DMA: 9,10-dimethylanthracene

Previous studies have found that the incorporation of nitrogen and carbon atoms into a polydentate ligand framework significantly influences the optical and electronic properties of the resulting complex, highlighting the potential applications of these species in the fields of biomedical and optoelectronic materials^7,10,11,13^. We therefore embarked on photophysical studies on four structurally well-defined polydentate chelates, **2**, **3**, **4**, and **5**. The ultraviolet/visible (UV/Vis) absorption spectra of N/C-based π systems **2**–**5** are summarized in Fig. 6a. These complexes each exhibit a broad absorption band ranging from the UV to the Vis region and extending into the near-infrared (NIR) region. Considering the low-energy absorption bands, the absorption maximum of NCCCN core complex **5** in the Vis region (*λ*_max_ = 669 nm) is redshifted by 194 nm compared with that of CCCCN core complex **2** (*λ*_max_ = 475 nm). The absorption peaks of non-aromatic NCCC complex **3** (*λ* = 409 nm, *ε* = 7.52 × 10^3^ M^−1^ cm^−1^ and *λ* = 754 nm, *ε* = 1.28 × 10^3^ M^−1^ cm^−1^) are similar to those of aromatic NCCC complex **4** (*λ* = 422 nm, *ε* = 9.57 × 10^3^ M^−1^ cm^−1^ and *λ* = 726 nm, *ε* = 2.24 × 10^3^ M^−1^ cm^−1^), but with a much smaller molar absorption coefficients, most likely due to its localized structure.

In particular, the effective low-energy absorptions of complexes **4** and **5** motivated us to study PA imaging properties^45,46^ of these metallaaromatic compounds. PA imaging is an emerging noninvasive molecular imaging technique based on the PA effect, which refers to the generation of acoustic waves due to thermal expansion following the absorption of light by absorbers. Compared with traditional optical imaging methods, PA imaging while retaining high spatial resolution remarkably enhances the penetration depth. A number of contrast agents, such as small molecule organic dyes, fluorescent proteins, metallic nanoparticles, organic nanoparticles, carbon nanotubes, 2D graphene analogs, porphysomes, and a few organometallic materials^47–49^, have been exploited in PA imaging. PA imaging of complexes **4** and **5** were performed in vitro using a PA instrument at *λ* = 700 nm. As shown in Fig. 6b, as expected, the PA signal increased with increasing concentrations of **4** and **5**. NCCCN core complex **5** exhibits a stronger PA signal than NCCC core complex **4**, which is probably due to the higher absorptivity of **5** compared to that of **4** at *λ* = 700 nm. Due to the relatively strong absorption of **4** at 808 nm, the photothermal effect^50^ was also examined by measuring the temperature increase in solution of **4** at various concentrations in water-ethanol (90% v/v) solutions under NIR laser (808 nm, 1 W cm^−2^) irradiation. As shown in Supplementary Figure 16, with increasing concentration of **4**, significant temperature increases were observed under laser irradiation. The solution containing 0.500 mg ml^−1^ of **4** exhibits a significant temperature increase from 32 to 78 °C within 5 min.

The unique π-conjugated structures of complexes **4** and **5** motivated us to further investigate their potential applicability as sonosensitizers in sonodynamic therapy (SDT). SDT employs US to activate sonosensitizers to generate reactive oxygen species (ROS), which are cytotoxic and can be used to suppress the growth of tumors and pathogenic bacteria. Various organic and inorganic sonosensitizers have been adopted for SDT^51–53^. However, to the best of our knowledge, the study of sonodynamic effects based on organometallics has never been reported. Since ROS generation plays a critical role in determining the therapeutic efficacy of SDT, the ability of the complexes to generate ROS was therefore investigated. As shown in Fig. 6c, d, with increasing concentration, the fluorescence (FL) intensity of 9,10-dimethylanthracene (DMA) decreased, indicating that complexes **4** and **5** had the ability to generate ROS under ultrasound. Combined with their good performance of PA imaging, aromatic NCCC complex **4** and NCCCN complex **5** can be regarded as potential theranostic agents for PA imaging-guided SDT.

In summary, we have synthesized a series of planar, high-coordinate N/C complexes from reactions of CCCC tetradentate precursors. The pentadentate chelates have now been extended to the equatorial N/C joint coordination environment to build pentadentate complexes with CCCCN and NCCCN cores. Our studies revealed that the key to the success of this approach is the rich reactivity of the carbon-based ligands in accordance with the inherent aromaticity of the polycyclic metallaaromatic structures. Furthermore, the direct transfer of coordinating atoms in high-coordinate complexes allows a number of exceptional transformations, such as the migration of the metallaaromatic unit within the polycyclic system, the alteration of the aromatic ring skeleton, and the modification of the aromaticity of the aza-metallacyclic rings. Our findings provide a valuable supplement to the dearth of information available regarding high-coordinate complexes and hint at efficient route towards pentadentate complexes with N/C joint coordination sites. The unique examples reported herein exhibit significant sonodynamic effects and good PA performances, demonstrating their promise as theranostic agents for PA imaging-guided SDT for cancer or bacterial therapies.

## Methods

### General methods

All syntheses were carried out under an inert atmosphere (N_2_) by means of standard Schlenk techniques, unless otherwise stated. Solvents were distilled from sodium/benzophenone (hexane and diethyl ether) or calcium hydride (dichloromethane) under N_2_ prior to use. Reagents were used as received from commercial sources without further purification. NMR spectroscopic experiments were performed on a Bruker Ascend 600 spectrometer (^1^H, 600.1 MHz; ^13^C, 150.9 MHz; ^31^P, 242.9 MHz) at room temperature and a Bruker AV-500 spectrometer (^1^H, 500.2 MHz; ^13^C, 125.8 MHz; ^31^P, 202.5 MHz) at room temperature. ^1^H and ^13^C NMR chemical shifts (δ) are relative to tetramethylsilane, and ^31^P NMR chemical shifts are relative to 85% H_3_PO_4_. The absolute values of the coupling constants are given in hertz (Hz). Multiplicities are abbreviated as singlet (s), doublet (d), triplet (t), multiplet (m), quartet (q), and broad (br). The high-resolution mass spectrometry (HRMS) data were recorded on a Bruker En Apex Ultra 7.0 T FT-MS. The theoretical molecular ion peak was calculated by Compass Isotope Pattern software supplied by Bruker Co. For the HRMS, ^1^H, ^31^P NMR, and ^13^C NMR spectra of the complexes in this article, see Supplementary Figures 17–32. Elemental analysis data were obtained on an Elementar Analysensysteme GmbH Vario EL III instrument. The UV/Vis/NIR spectra of complexes **2**, **3**, **4**, and **5** were recorded with a Varian Cary5000 UV/Vis spectrophotometer.

### Synthesis of the starting material **1**

According to a previously published procedure^30^, allenylboronic acid pinacol ester (144 μl, 0.80 mmol) was added to a suspension of [OsCl_2_(CHC(PPh_3_)CH(OH)C≡CH)(PPh_3_)_2_] (300 mg, 0.27 mmol) in dichloromethane (15 ml). The mixture was stirred at room temperature (RT) for 30 min to give a yellow solution. The solution was evaporated under vacuum to a volume of ca. 2 ml, then diethyl ether (20 ml) was added to the solution. The yellow precipitate was collected by filtration, washed with diethyl ether (2 × 5 ml) and dried under vacuum to give **1** (275 mg, 89%) as a yellow solid.

### Synthesis of the complex with a CCCCN-core (**2**)

A mixture of **1** (200 mg, 0.174 mmol), 2-ethynylpyridine (52 μl, 0.522 mmol), and silver perchlorate (144 mg, 0.696 mmol) in dichloromethane/methanol (9/3 ml) was stirred at 60 °C in a sealed Schlenk tube for 1 h to give an amaranthine solution. The silver chloride precipitate was removed by filtration, and the filtrate was concentrated under vacuum to ~2 ml of residue. The residue was washed with Et_2_O (2 × 15 ml) to give **2** as an amaranthine solid. Yield: 223 mg, 92%. Diagnostic peaks for **2** are as follows: ^1^H NMR plus ^1^H-^13^C HSQC (600.1 MHz, CD_2_Cl_2_): *δ* = 14.2 (d, ^3^*J*_HP_ = 14.3 Hz, 1H, C^1^*H*), 9.7 (d, ^4^*J*_HP_ = 3.6 Hz, 1H, C^5^*H*), 9.1 (dd, ^3^*J*_HP_ = 3.8 Hz, ^4^*J*_PH_ = 2.3 Hz, 1 H, C^3^*H*), 8.8 (d, ^3^*J*_HH_ = 5.3 Hz, 1H, C^15^*H*), 8.1 (m, 1H, C^6^*H*), 7.7–6.6 (48H, other aromatic protons), 6.5 ppm (d, ^4^*J*_HP_ = 7.6 Hz, 1H, C^9^*H*). ^31^P NMR (242.9 MHz, CD_2_Cl_2_): *δ* = 12.1 (s, C*P*Ph_3_), –8.6 ppm (s, Os*P*Ph_3_). ^13^C NMR plus ^1^H-^13^C HMBC and ^1^H-^13^C HSQC (150.9 MHz, CD_2_Cl_2_): δ = 232.4 (br, C1), 220.9 (t, ^2^*J*_CP_ = 9.0 Hz, C7), 206.9 (s, C8), 198.4 (dt, ^2^*J*_CP_ = 23.5 Hz, ^3^*J*_CP_ = 3.7 Hz, C4), 169.6 (s, C5), 168.9 (s, C11), 166.7 (d, ^2^*J*_CP_ = 20.4 Hz, C3), 162.5 (t, ^2^*J*_CP_ = 7.8 Hz, C10), 157.0 (s, C6), 145.0 (s, C15), 141.6 (d, ^1^*J*_PC_ = 70.4 Hz, C2), 119.8 (s, C9), 140.7–116.6 ppm (other aromatic carbons and the above-mentioned C9). Elemental analysis calcd (%) for C_69_H_54_Cl_2_NO_9_OsP_3_: C 59.40, H 3.90, N 1.00; found: C 59.19, H 4.03, N 1.36. HRMS (ESI): *m/z* calcd for [C_69_H_54_NOOsP_3_]^2+^, 598.6515; found, 598.6529.

### Synthesis of the complex with an NCCC-core (**3**)

A mixture of **2** (200 mg, 0.143 mmol) and azidotrimethylsilane (94 μl, 0.72 mmol) was stirred at room temperature for 12 h to give a green solution. Then, *n*-Bu_4_NF (0.72 ml, 1.0 mol l^−1^ in tetrahydrofuran, 0.72 mmol) was added to the solution. The mixture was stirred at room temperature for 1 h and then concentrated under vacuum to ~2 ml. The residue was purified by column chromatography (neutral alumina, eluent: dichloromethane/methanol = 20:1) to give a green solution. The solvent was evaporated to under vacuum to give ~2 ml of residue, which was then washed with Et_2_O (2 × 15 ml) to give **3** as a green solid. Yield: 107 mg, 57%. Diagnostic peaks for **3** are as follows: ^1^H NMR plus ^1^H-^13^C HSQC (500.2 MHz, CD_2_Cl_2_): *δ* = 8.5 (d, ^3^*J*_HH_ = 3.6 Hz, 1H, C^15^*H*), 7.5 (s, 1H, C^6^*H*), 7.4 (s, 1H, C^9^*H*), 7.8–6.9 (49H, other aromatic protons and the above-mentioned C^6^*H* and C^9^*H*), 6.5 (d, ^3^*J*_HH_ = 8.1 Hz, 1H, C^12^*H*), 5.9 (s, 1H, C^3^*H*), 5.4 (s, 1H, C^5^*H*), 4.5 ppm (d, ^3^*J*_PH_ = 15.9 Hz, 1 H, C^1^*H*). ^31^P NMR (202.5 MHz, CD_2_Cl_2_): *δ* = 18.0 (s, C*P*Ph_3_), 3.0 ppm (s, Os*P*Ph_3_). ^13^C NMR plus ^1^H-^13^C HMBC and ^1^H-^13^C HSQC (125.8 MHz, CD_2_Cl_2_): *δ* = 218.8 (t, ^2^*J*_CP_ = 7.6 Hz, C7), 197.0 (br, C10), 196.1 (s, C8), 177.2 (dt, ^2^*J*_CP_ = 19.2 Hz, ^3^*J*_CP_ = 9.1 Hz, C4), 166.1 (br, C5), 164.5 (s, C11), 159.5 (d, ^2^*J*_CP_ = 13.0 Hz, C1), 147.8 (s, C15), 142.4 (s, C9), 138.6 (s, C6), 137.5 (d, ^2^*J*_CP_ = 15.6 Hz, C3), 119.2 (s, C12), 136.3–117.3 (other aromatic carbons and the above-mentioned C12), 93.0 ppm (d, ^1^*J*_PC_ = 91.5 Hz, C2). Elemental analysis calcd (%) for C_69_H_54_ClN_2_O_5_OsP_3_: C 63.27, H 4.16, N 2.14; found: C 63.27, H 4.46, N 2.09. HRMS (ESI): *m*/*z* calcd for [C_69_H_54_N_2_OOsP_3_]^+^, 1211.3065; found, 1211.3034.

### Synthesis of the complex with an NCCC-core (**4**)

A mixture of **3** (100 mg, 76.3 μmol) and MeOTf (8.6 μl, 76.3 μmol) in dichloromethane (10 ml) was stirred at room temperature for 15 min to give a green solution. The solvent was evaporated under vacuum to give ~2 ml of residue. The residue was washed with Et_2_O (2 × 15 ml) to give **4** as a green solid. Yield: 102 mg, 91%. Diagnostic peaks for **4** are as follows: ^1^H NMR plus ^1^H-^13^C HSQC (500.2 MHz, CD_2_Cl_2_): *δ* = 8.9 (s, 1H, C^15^*H*), 8.8 (s, 1H, C^6^*H*), 8.3 (s, 1H, C^9^*H*), 7.0 (s, 1H, C^5^*H*), 7.0 (s, 1H, C^3^*H*), 7.8–6.9 (50 H, other aromatic protons and the above-mentioned C^3^*H* and C^5^*H*), 5.6 (d, ^3^*J*_HP_ = 14.2 Hz, 1 H, C^1^*H*), 3.8 ppm (s, 3 H, C^16^*H*). ^31^P NMR (202.5 MHz, CD_2_Cl_2_): *δ* = 19.6 (s, C*P*Ph_3_), –3.2 ppm (s, Os*P*Ph_3_). ^13^C NMR plus ^1^H-^13^C HMBC and ^1^H-^13^C HSQC (125.8 MHz, CD_2_Cl_2_): *δ* = 248.3 (t, ^2^*J*_CP_ = 7.5 Hz, C10), 193.4 (dt, ^2^*J*_CP_ = 18.6 Hz, ^3^*J*_CP_ = 9.9 Hz, C4), 178.3 (s, C7), 175.6 (s, C8), 163.7 (br, C5), 162.9 (s, C11), 156.3 (d, ^2^*J*_CP_ = 12.9 Hz, C1), 150.0 (s, C6), 149.8 (s, C15), 140.3 (d, ^2^*J*_CP_ = 15.7 Hz, C3), 137.9 (s, C9), 121.0 (q, J(F,C) = 317.7 Hz, CF_3_SO_3_), 137.2–116.0 (other aromatic carbons and the above-mentioned CF_3_SO_3_), 98.1 (d, ^1^*J*_CP_ = 88.7 Hz, C2), 60.5 ppm (s, C16). Elemental analysis calcd (%) for C_71_H_57_ClF_3_N_2_O_8_OsP_3_S: C 57.86, H 3.90, N 1.90; found: C 57.87, H 4.10, N 1.77. HRMS (ESI): *m*/*z* calcd for [C_70_H_57_N_2_OOsP_3_]^2+^, 613.1647; found, 613.1631.

### Synthesis of the complex with an NCCCN-core (**5**)

A mixture of **4** (100 mg, 67.8 μmol) and perchloric acid (29 μl, 70 wt%) in dichloromethane (10 ml) was stirred at room temperature for 1 h to give a deep green solution. The solvent was evaporated under vacuum to give ~2 ml of crude material. The residue was washed with Et_2_O (1 × 15 ml) to give **5** as a deep green solid. Yield: 94 mg, 88%. Diagnostic peaks for **5** are as follows: ^1^H NMR plus ^1^H-^13^C HSQC (500.2 MHz, CD_2_Cl_2_): *δ* = 11.3 (d, ^3^*J*_HP_ = 11.9 Hz, 1H, N*H*), 9.3 (dd, ^3^*J*_HP_ = 11.6 Hz, ^4^*J*_HP_ = 5.3 Hz, 1H, C^1^*H*), 8.9 (d, ^3^*J*_HH_ = 5.0 Hz, 1H, C^15^*H*), 8.8 (t, ^4^*J*_HP_ = 1.9 Hz, 1H, C^9^*H*), 8.4 (d, ^4^*J*_HP_ = 3.4 Hz, 1H, C^6^*H*), 8.3 (dd, ^4^*J*_HP_ = 6.2 Hz, ^4^*J*_HP_ = 3.2 Hz, 1H, C^5^*H*), 7.6 (d, ^3^*J*_HP_ = 11.6 Hz, ^4^*J*_HP_ = 3.7 Hz, 1H, C^3^*H*), 6.7–7.9 (49H, other aromatic protons and the above-mentioned C^3^*H*), 4.0 ppm (s, 3H, C^16^*H*). ^31^P NMR (202.5 MHz, CD_2_Cl_2_): *δ* = 22.3 (s, C*P*Ph_3_), –14.2 ppm (s, Os*P*Ph_3_). ^13^C NMR plus ^1^H-^13^C HMBC and ^1^H-^13^C HSQC (125.8 MHz, CD_2_Cl_2_): *δ* = 189.4 (s, C10), 185.6 (s, C8), 184.8 (m, C4), 183.4 (m, C7), 175.3 (s, C5), 165.8 (s, C11), 165.5 (d, ^2^*J*_CP_ = 17.6 Hz, C1), 161.7 (d, ^2^*J*_CP_ = 7.9 Hz, C3), 155.1 (s, C6), 143.6 (s, C15), 132.4 (s, C9), 119.9 (q, J(F,C) = 317.4 Hz, CF_3_SO_3_), 141.8–114.1 (other aromatic carbons and the above-mentioned C9 and CF_3_SO_3_), 109.1 (d, ^1^*J*_CP_ = 97.4 Hz, C2), 62.6 ppm (s, C16). Elemental analysis calcd (%) for C_71_H_58_Cl_2_F_3_N_2_O_12_OsP_3_S: C 54.17, H 3.71, N 1.78; found: C 54.20, H 3.97, N 1.43. HRMS (ESI): *m*/*z* calcd for [C_70_H_58_N_2_OOsP_3_^3+^-H^+^]^2+^, 613.1647; found, 613.1663.

### PA imaging

Solutions of **4** and **5** at different concentrations in MeCN were stored in 0.2-ml transparent plastic vials. Then, PA images of **4** and **5** were obtained at 700 nm using a PA system (Endra Nexus 128, Ann Arbor, MI, USA).

### Photothermal effect

Solutions of complex **4** at different concentrations in 1 ml of 10% ethanol-H_2_O were placed in transparent plastic vials. Then, the solutions were irradiated with an 808 nm laser at a power density of 1 W cm^−2^ for 10 min. The temperature was monitored by a digital thermometer. The real-time temperature changes were recorded by a FLIR Ax5 thermal camera.

### Assessment of ROS generation by **4**/**5** upon sono-activation

The production of ROS was examined based on the decrease in the FL of DMA, an ROS sensor. Solutions of **4** and **5** in DCM were prepared at different concentrations. DMA was then added to give a final concentration of 20 μM. Each sample was exposed to ultrasound (1 MHz, 0.56 Wcm^−2^, 50% cycle, 5 min). The FL intensity of the DMA was detected at and excitation wavelength of 360 nm and an emission range of 380–550 nm.

### Computational details

All structures were optimized at the B3LYP level of DFT^54–56^ with the PCM solvation method in dichloromethane. Additionally, frequency calculations were performed to confirm that the energies of the calculated structures were at minima (zero imaginary frequency) or transition states (one imaginary frequency), and to provide Gibbs free energies at 298.15 K. In the B3LYP calculations, the effective core potentials given by Hay and Wadt with a double-ζ valence basis set (LanL2DZ)^57^ were used to describe the Os, Cl, Si, and P atoms, whereas the standard 6-311++G** basis set was used for the C, N, O, and H atoms for all the model compounds in the ASE, NICS, ACID, and Wiberg bond index calculations. For the mechanism study, we used the original structures without changing the PPh_3_ groups to PH_3_ groups to obtain a more reasonable estimation of the energy differences. All of these structures evaluated were optimized at the standard 6-31 G* basis set for C, N, O, and H atoms. Intrinsic reaction coordinate calculations were also calculated for the transition state to confirm that such structure indeed connected two relevant minima^58^. Polarization functions were added for Os (ζ(f) = 0.886), Cl (ζ(d) = 0.514), Si (ζ(d) = 0.262), and P (ζ(d) = 0.340)^59^ in all calculations. All optimizations were performed with the Gaussian 09 software package^60^. Wiberg bond index^61^ calculations were carried out with the NBO 6.0 program^62^ interfaced with the Gaussian 09 program. (NICS)^37–39^ values were calculated at the B3LYP-GIAO/6-311++G** level of theory. The ACID calculations were carried out with the ACID program^40,41^.

### X-ray crystallographic analysis

Crystals suitable for X-ray diffraction were grown from a dichloroethane solution (for **2**) or a dichloromethane solution (for **3**, **4**, and **5**) layered with hexane. Single-crystal X-ray diffraction data were collected on an Oxford Gemini S Ultra CCD area detector with graphite-monochromated Mo Kα radiation (*λ* = 0.71073 Å) for **2**, **4**, and **5**. An Agilent SuperNova Dual system with mirror-monochromated Mo Kα radiation (*λ* = 0.71073 Å) was used for **3**. Using Olex2^63^, the structures were solved using the SHELXT^64^ structure solution program using the intrinsic phasing method (**2**, **3**, **4**, and **5**), and all of the structures were refined with the SHELXL^65^ refinement package using least-squares minimization. All non-hydrogen atoms were refined anisotropically, unless otherwise stated. The hydrogen atoms were placed at their idealized positions and assumed the riding model, unless otherwise stated. The water (H_2_O) and dichloromethane (CH_2_Cl_2_) solvent molecules in **4** were refined without the addition of H atoms. For further details on the crystal data, data collection, refinements, and response to the questions raised in the Check CIF Reports, see Supplementary Table 1 and Supplementary Table 2.

## Supplementary information

Supplementary Information

Description of Additional Supplementary Files

Supplementary Data

## Data Availability

The authors declare that the main data supporting the findings of this study are available within the article and its Supplementary Information file and Supplementary Data file. Crystallographic data are available through the Cambridge Crystallographic Data Center: CCDC identifiers are CCDC 1869552 (complex **2**), CCDC 1869553 (complex **3**), CCDC 1869554 (complex **4**), and CCDC 1869556 (complex **5**). Extra data are available from the corresponding author upon request.
