# Supplementary material for: Successive modification of polydentate complexes gives access to planar carbon- and nitrogen-based ligands
Source: Nat Commun. 2019 Apr 2;10:1488. doi: 10.1038/s41467-019-09367-8 (PMC6445293; doi:10.1038/s41467-019-09367-8)
Supplement: Supplementary file 1 — Supplementary Information [file 41467_2019_9367_MOESM1_ESM.pdf]

Successive modification of polydentate complexes gives access to planar carbon- and nitrogen-based ligands

Zhou et al.

Supplementary Figures and Tables

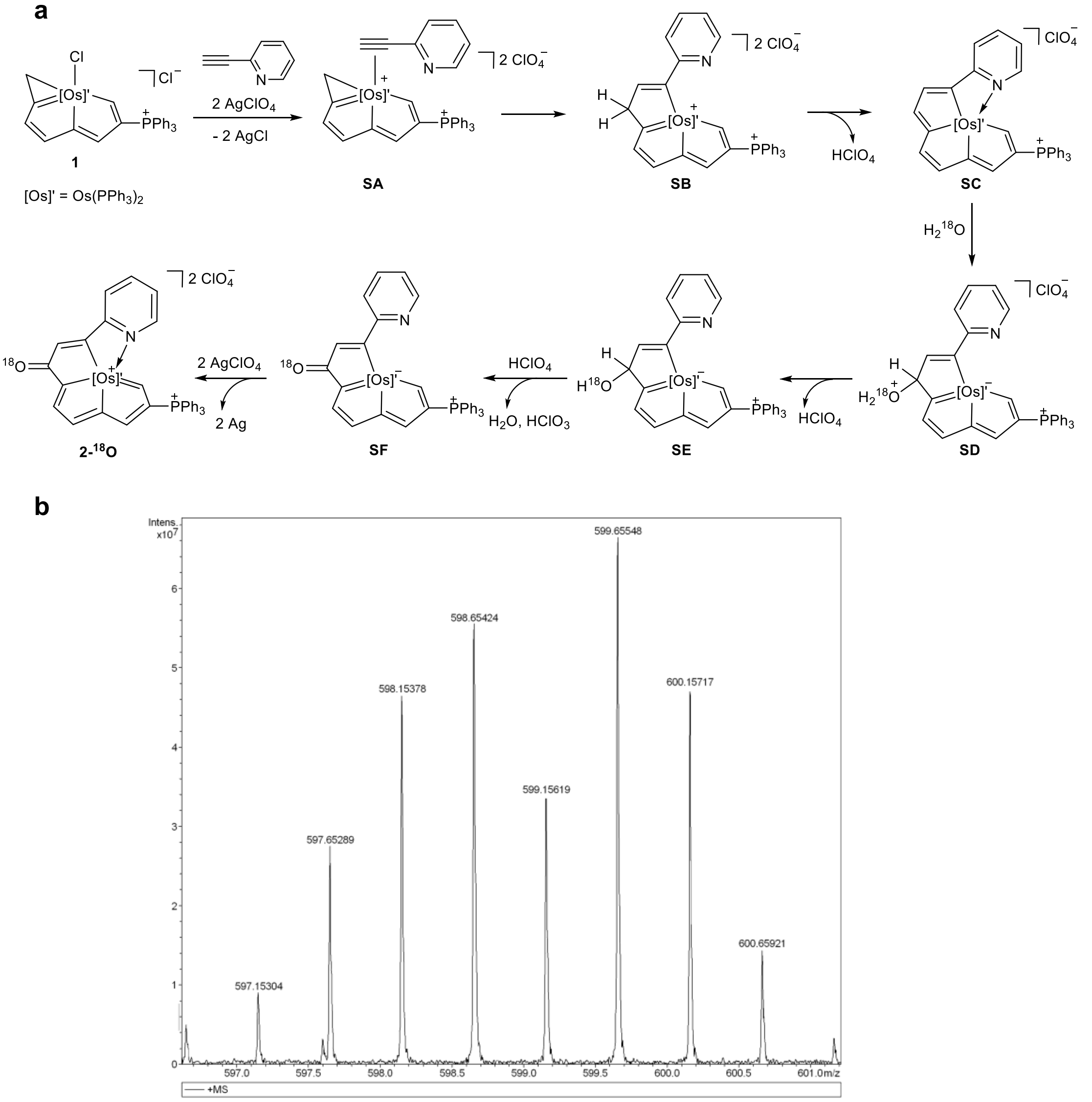

Supplementary Figure 1 | Proposed mechanism for the synthesis of complex 2 and ^{18}O-labeling experiment. a) Proposed mechanism for the formation of complex **2**. **b**) positive-ion ESI-MS spectrum of [**2-^{18}O**]$^{2+}$ [$C_{69}H_{54}N^{18}OOsP_3$]$^{2+}$ measured in methanol. The experimental procedures of the ^{18}O-labeling experiment: A mixture of **1** (10 mg, 8.69 μmol), 2-ethynylpyridine (2.6 μl, 26.1 μmol), silver perchlorate (7.2 mg, 34.8 μmol) and excess $H_2{}^{18}O$ (5 μl) in dichloromethane/methanol (0.3/0.1 ml) was heated at 60 °C in a sealed NMR tube for 1 h to give a purple red solution. The *in situ* yield of complex **2-^{18}O** is about 90%, as suggested by ^{1}H- and ^{31}P-NMR. HRMS (ESI): m/z calcd for [$C_{69}H_{54}N^{18}OOsP_3$]$^{2+}$, 599.6536; found, 599.6555.

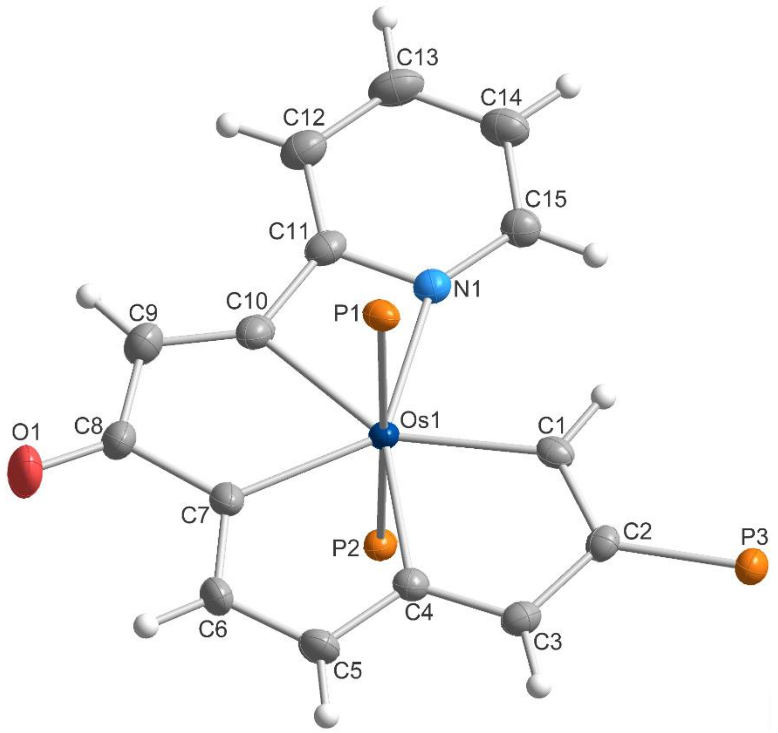

Supplementary Figure 2 | X-ray molecular structure for the cation of complex **2** drawn with 50% probability. Phenyl groups in PPh_3 moieties were omitted for clarity. Selected bond lengths [Å] and angles [deg]: Os1−C1 2.046(5), Os1−C4 2.103(5), Os1−C7 2.088(5), Os1−C10 2.117(5), Os1−N1 2.232(4), C1−C2 1.390(7), C2−C3 1.403(7), C3−C4 1.390(7), C4−C5 1.402(7), C5−C6 1.383(8), C6−C7 1.366(8), C7−C8 1.515(7), C8−C9 1.445(8), C9−C10 1.336(8), C10−C11 1.451(8), C11−N1 1.359(7), C8−O1 1.235(7); Os1−C1−C2 122.0(4), C1−C2−C3 112.5(5), C2−C3−C4 112.8(5), C3−C4−Os1 119.7(4), C4−Os1−C1 73.1(2), Os1−C4−C5 120.2(4), C4−C5−C6 112.5(5), C5−C6−C7 113.3(5), C6−C7−Os1 121.9(4), C7−Os1−C4 72.0(2), Os1−C7−C8 120.5(4), C7−C8−C9 110.7(5), C8−C9−C10 110.0(5), C9−C10−Os1 127.7(4), C10−Os1−C7 71.1(2), Os1−C10−C11 99.2(3), C10−C11−N1 103.0(4), C11−N1−Os1 97.0(3), N1−Os1−C10 60.72(19).

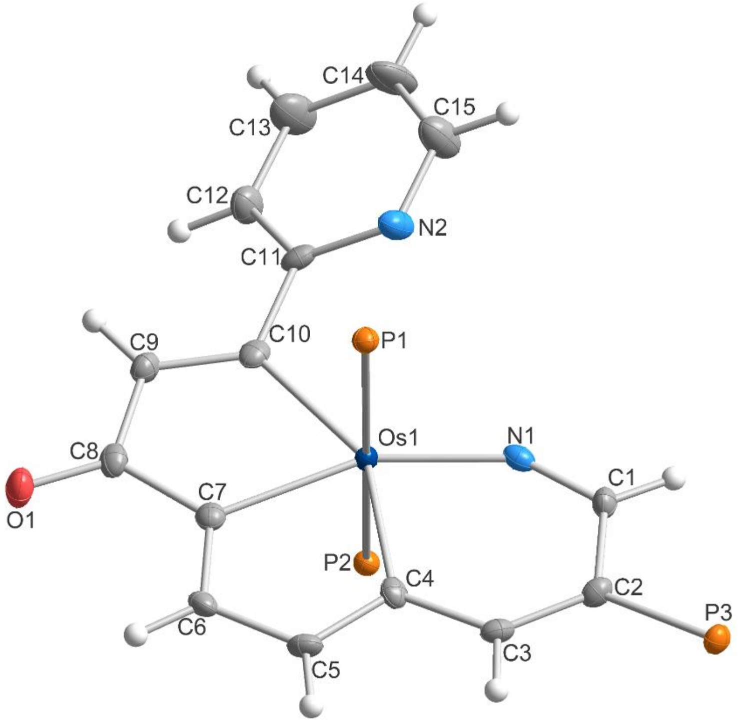

Supplementary Figure 3 | X-ray molecular structure for the cation of complex **3** drawn with 50% probability. Phenyl groups in PPh_3 moieties were omitted for clarity. Selected bond lengths [Å] and angles [deg]: Os1−N1 1.891(3), Os1−C4 2.102(4), Os1−C7 2.123(4), Os1−C10 2.090(4), N1−C1 1.270(5), C1−C2 1.470(5), C2−C3 1.367(5), C3−C4 1.432(5), C4−C5 1.375(5), C5−C6 1.421(5), C6−C7 1.348(5), C7−C8 1.474(5), C8−C9 1.449(6), C9−C10 1.367(5), C10−C11 1.476(5), C8−O1 1.235(4); Os1−N1−C1 144.6(3), N1−C1−C2 120.2(3), C1−C2−C3 119.2(3), C2−C3−C4 127.7(4), C3−C4−Os1 125.9(3), C4−Os1−N1 82.06(13), Os1−C4−C5 117.6(3), C4−C5−C6 115.3(4), C5−C6−C7 113.9(3), C6−C7−Os1 118.8(3), C7−Os1−C4 74.38(14), Os1−C7−C8 119.6(3), C7−C8−C9 109.8(3), C8−C9−C10 114.4(4), C9−C10−Os1 122.7(3), C7−Os1−C10 73.43(15).

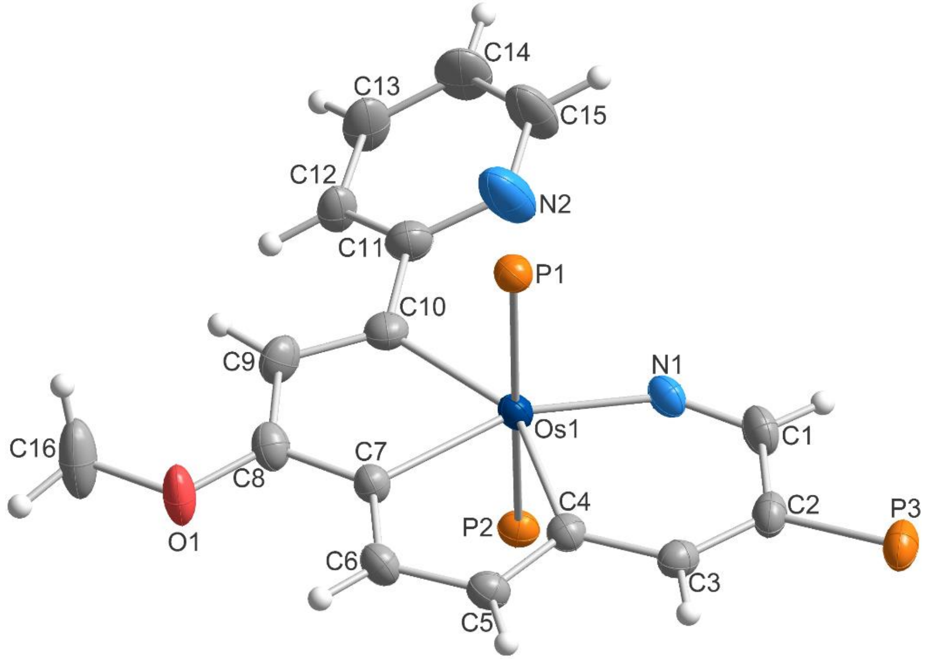

Supplementary Figure 4 | X-ray molecular structure for the cation of complex **4** drawn with 50% probability. Phenyl groups in PPh_3 moieties were omitted for clarity. Selected bond lengths [Å] and angles [deg]: Os1−N1 1.879(4), Os1−C4 2.092(4), Os1−C7 2.110(4), Os1−C10 2.067(5), N1−C1 1.267(6), C1−C2 1.463(7), C2−C3 1.347(6), C3−C4 1.443(6), C4−C5 1.372(6), C5−C6 1.401(6), C6−C7 1.371(6), C7−C8 1.410(6), C8−C9 1.391(6), C9−C10 1.378(6), C10−C11 1.472(6), C8−O1 1.334(5), O1−C16 1.457(6); Os1−N1−C1 145.9(3), N1−C1−C2 119.3(4), C1−C2−C3 119.4(4), C2−C3−C4 127.9(4), C3−C4−Os1 125.6(3), C4−Os1−N1 81.61(16), Os1−C4−C5 118.4(3), C4−C5−C6 115.6(4), C5−C6−C7 112.8(4), C6−C7−Os1 119.3(3), C7−Os1−C4 73.87(16), Os1−C7−C8 118.1(3), C7−C8−C9 113.8(4), C8−C9−C10 113.0(4), C9−C10−Os1 121.5(3), C7−Os1−C10 73.47(17).

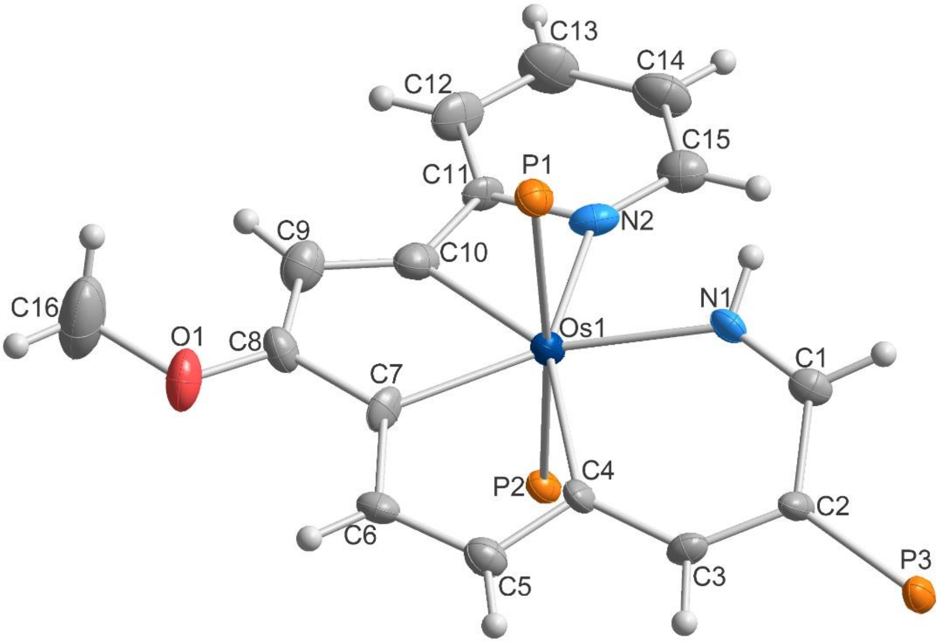

Supplementary Figure 5 | X-ray molecular structure for the cation of complex **5** drawn with 50% probability. Phenyl groups in PPh_3 moieties were omitted for clarity. Selected bond lengths [Å] and angles [deg]: Os1−N1 2.124(5), Os1−C4 2.098(6), Os1−C7 2.138(6), Os1−C10 2.066(6), Os1−N2 2.201(5), N1−C1 1.294(7), C1−C2 1.439(8), C2−C3 1.350(8), C3−C4 1.451(8), C4−C5 1.385(8), C5−C6 1.379(9), C6−C7 1.384(8), C7−C8 1.440(8), C8−C9 1.392(9), C9−C10 1.363(9), C10−C11 1.432(9), C11−N2 1.367(8), C8−O1 1.313(8), O1−C16 1.445(9); Os1−N1−C1 136.8(4), N1−C1−C2 122.4(5), C1−C2−C3 120.1(5), C2−C3−C4 129.5(6), C3−C4−Os1 125.7(4), C4−Os1−N1 80.9(2), Os1−C4−C5 120.1(4), C4−C5−C6 114.8(6), C5−C6−C7 112.9(6), C6−C7−Os1 119.7(4), C7−Os1−C4 72.3(2), Os1−C7−C8 119.2(4), C7−C8−C9 113.0(6), C8−C9−C10 109.7(6), C9−C10−Os1 127.3(5), C7−Os1−C10 70.0(2), C10−Os1−N2 61.6(2), C11−N2−Os1 95.7(4), C10−C11−N2 102.8(5).

Supplementary Table 1 | Crystal data and structure refinement for **2**, **3**, **4**, and **5**.

	**2**·$C_2H_4Cl_2$	2(**3**)·5(CH_2Cl_2)·0.5H_2O	**4**·0.5CH_2Cl_2·H_2O	**5**·CH_2Cl_2
Formular	$C_{71}H_{58}Cl_4NO_9OsP_3$	$C_{143}H_{118}Cl_{12}N_4O_{10.5}Os_2P_6$	$C_{72}H_{60}Cl_{1.5}F_{4.5}N_2O_{8.5}OsP_3S_{1.5}$	$C_{71.67}H_{60}Cl_{4.33}F_2N_2O_{12.33}OsP_3S_{0.67}$
Mr	1494.09	3052.03	1559.09	1642.65
Crystal system	Triclinic	Triclinic	Triclinic	Monoclinic
Space group	$P\bar{1}$	$P\bar{1}$	$P\bar{1}$	*I2/a*
a [Å]	11.7734(3)	10.52229(19)	12.0252(6)	20.2404(9)
b [Å]	14.2217(4)	23.2643(2)	14.6931(6)	18.8032(8)
c [Å]	20.1865(5)	26.9920(4)	20.6200(5)	41.011(3)
α [°]	85.170(2)	97.1893(9)	74.531(3)	90
β [°]	87.370(2)	94.6652(13)	73.562(3)	101.717(5)
γ [°]	89.810(2)	95.1433(11)	80.033(4)	90
V [Å^3]	3364.43(16)	6500.42(16)	3349.0(2)	15282.9(14)
Z	2	2	2	8
ρ_{calcd} [gcm^{-3}]	1.475	1.559	1.546	1.428
μ [mm^{-1}]	2.182	2.336	2.153	1.964
F (000)	1504.0	3068.0	1570.0	6608.0
2θ range [°]	3.374 to 52.5	3.322 to 49.998	3.552 to 52.5	2.986 to 58.388
Reflns collected	29793	58730	27356	83051
Independent reflns	13591	22910	13495	18475
Obserbed reflns [$I \geq 2\sigma(I)$]	12540	20225	12061	13774
Data/restrains/params	13591/32/829	22910/210/1655	13495/174/947	18475/97/938
GOF on F^2	1.048	1.044	1.072	1.046
R_1/wR_2 [$I \geq 2\sigma(I)$]	0.0479/0.1242	0.0346/0.0837	0.0428/0.1058	0.0712/0.1378
R_1/wR_2 (all data)	0.0530/0.1268	0.0412/0.0874	0.0506/0.1093	0.1025/0.1505
Largest peak/hole[e Å^{-3}]	1.66/-2.64	1.92/-1.20	1.75/-1.26	1.37/-1.57

Supplementary Table 2 | Response to the questions raised in the Check CIF Reports of complex **2**.

**Alert level B in Complex 2**	Hirshfeld Test (Solvent) Cl1 --O1AA . 16.0 s.u.	This problem is caused by the disorder of the ClO_4 anion.

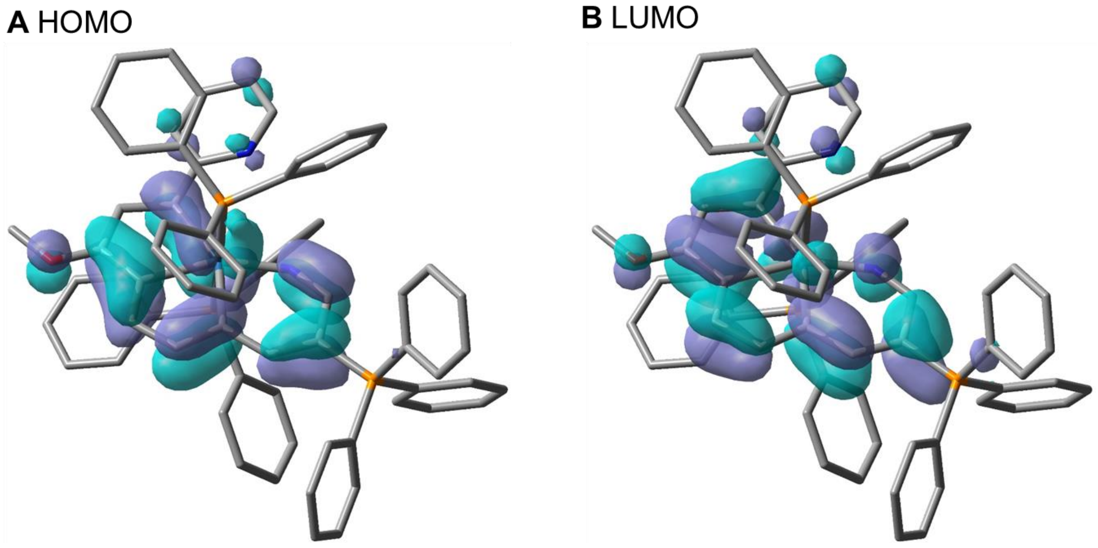

Supplementary Figure 6 | Orbital distributions in LUMO/ HOMO of complex **4**.

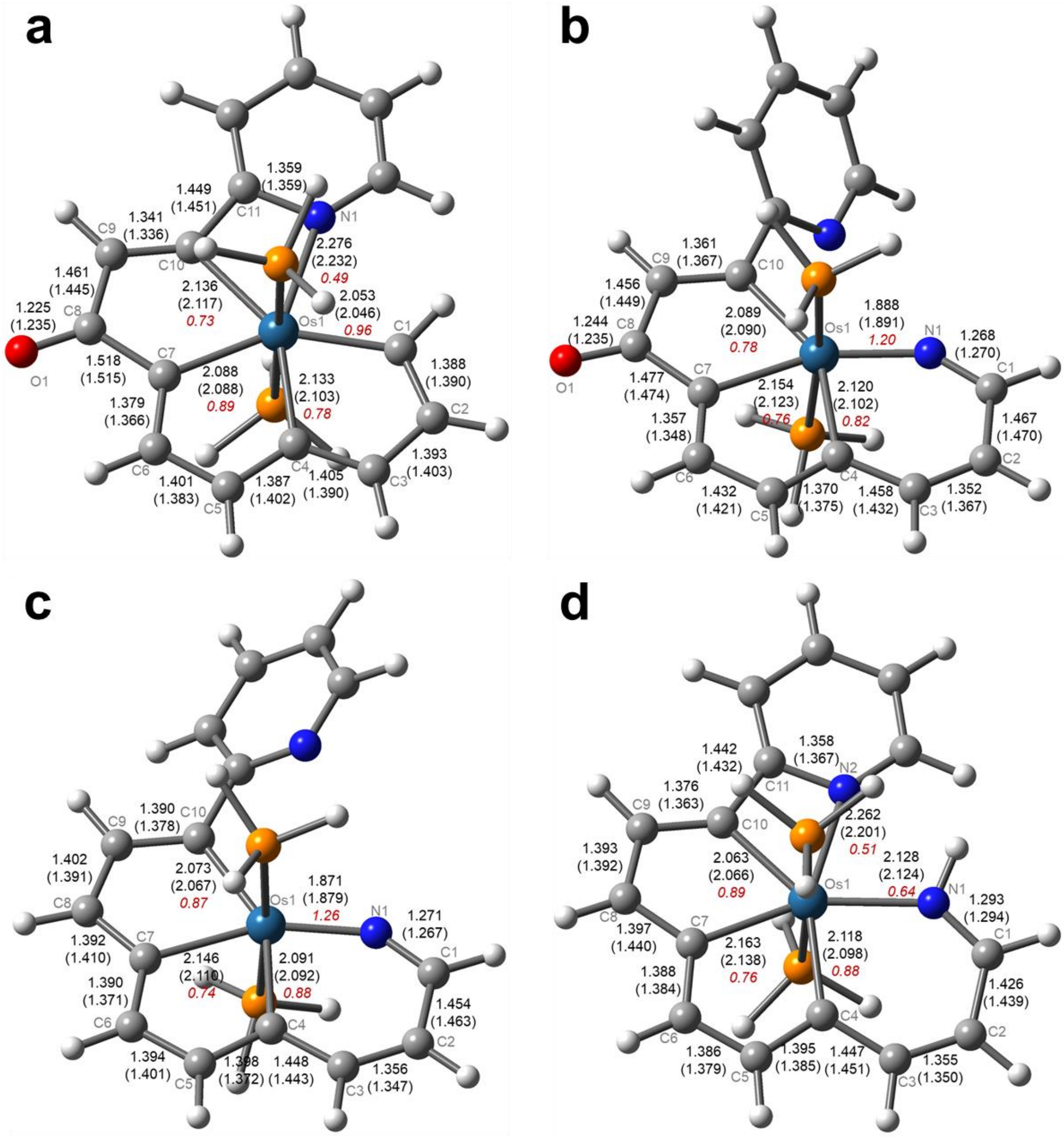

Supplementary Figure 7 | Comparison of the calculated and experimental (in parentheses) bond lengths (Å) for the cations of complexes **2**(a), **3**(b), **4**(c), and **5**(d). In the calculated structures, PH_3 was used to model PPh_3. The Wiberg bond indices of the bonds around the osmium center are shown in red in italics.

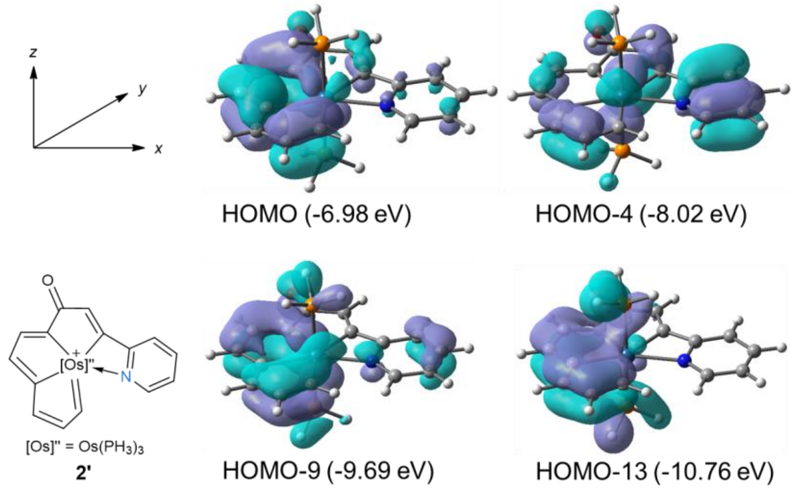

Supplementary Figure 8 | Four key occupied perimeter π molecular orbitals (π-MOs) of the osmapentalene unit in the model complex **2′**. The eigenvalues of the MOs are given in parentheses.

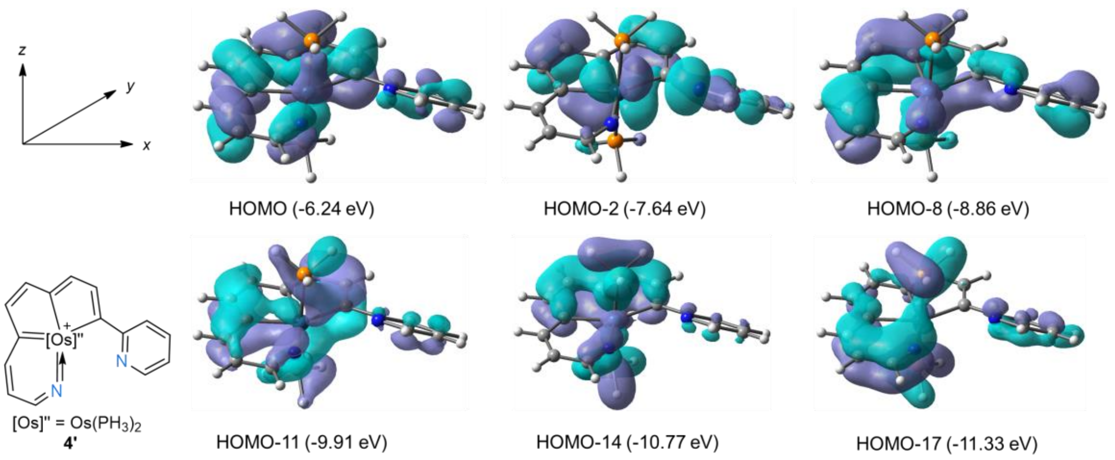

Supplementary Figure 9 | Six key occupied perimeter π molecular orbitals (π-MOs) of the osmapentalene fused pyridine unit in the model complex **4′**. The eigenvalues of the MOs are given in parentheses.

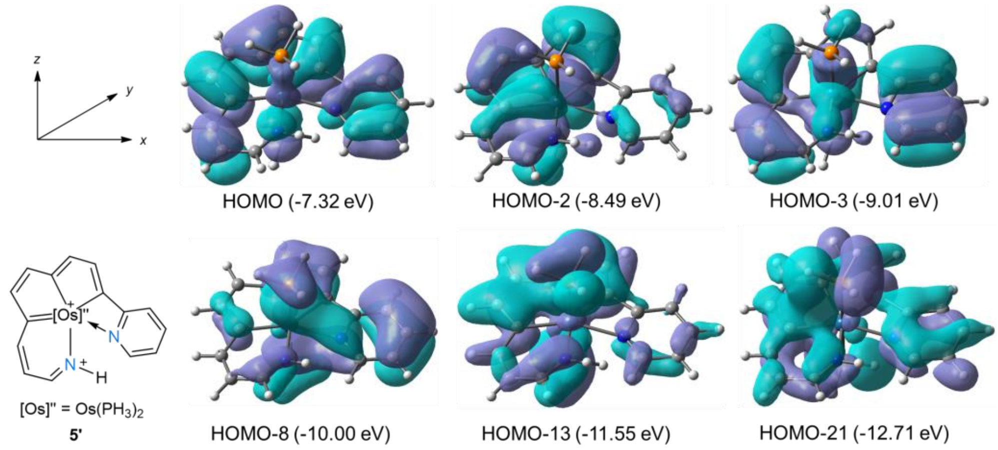

Supplementary Figure 10 | Six key occupied perimeter π molecular orbitals (π-MOs) of the osmapentalene fused pyridinium unit in the model complex **5′**. The eigenvalues of the MOs are given in parentheses.

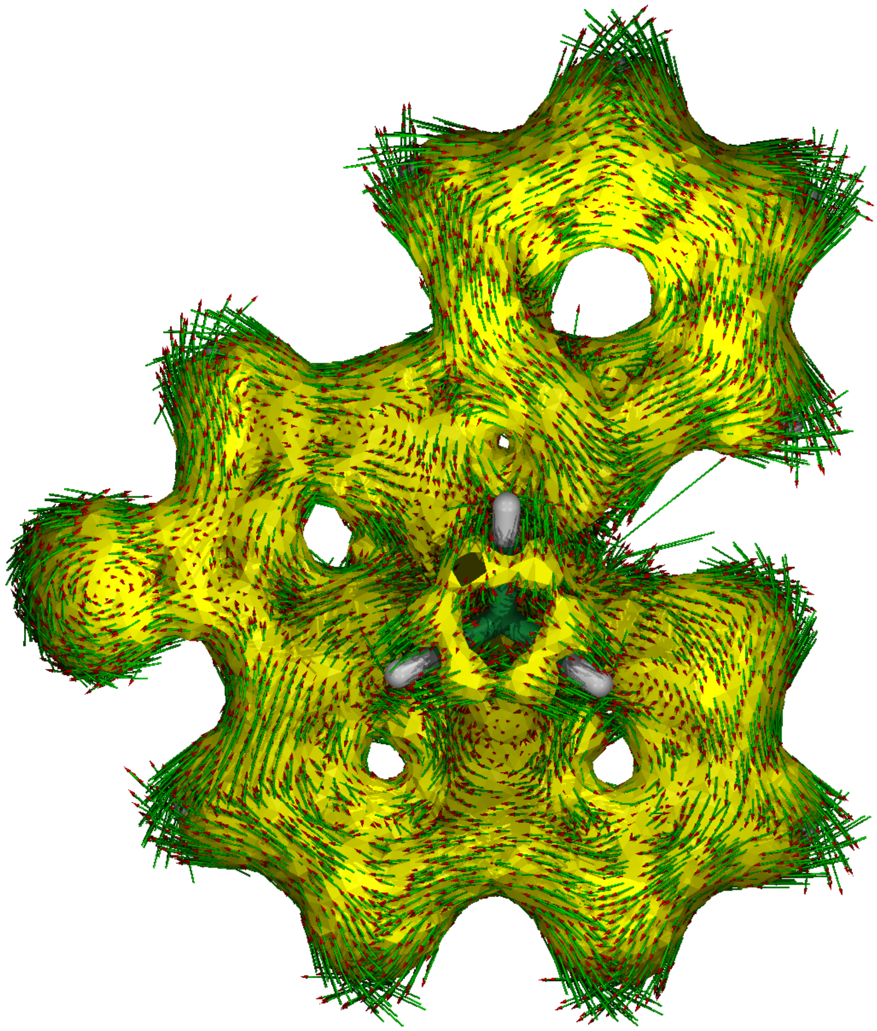

Supplementary Figure 11 | ACID plot of the model complex **2′** with an isosurface value of 0.025. The magnetic field vector is orthogonal to the ring plane and points upward (clockwise currents are diatropic).

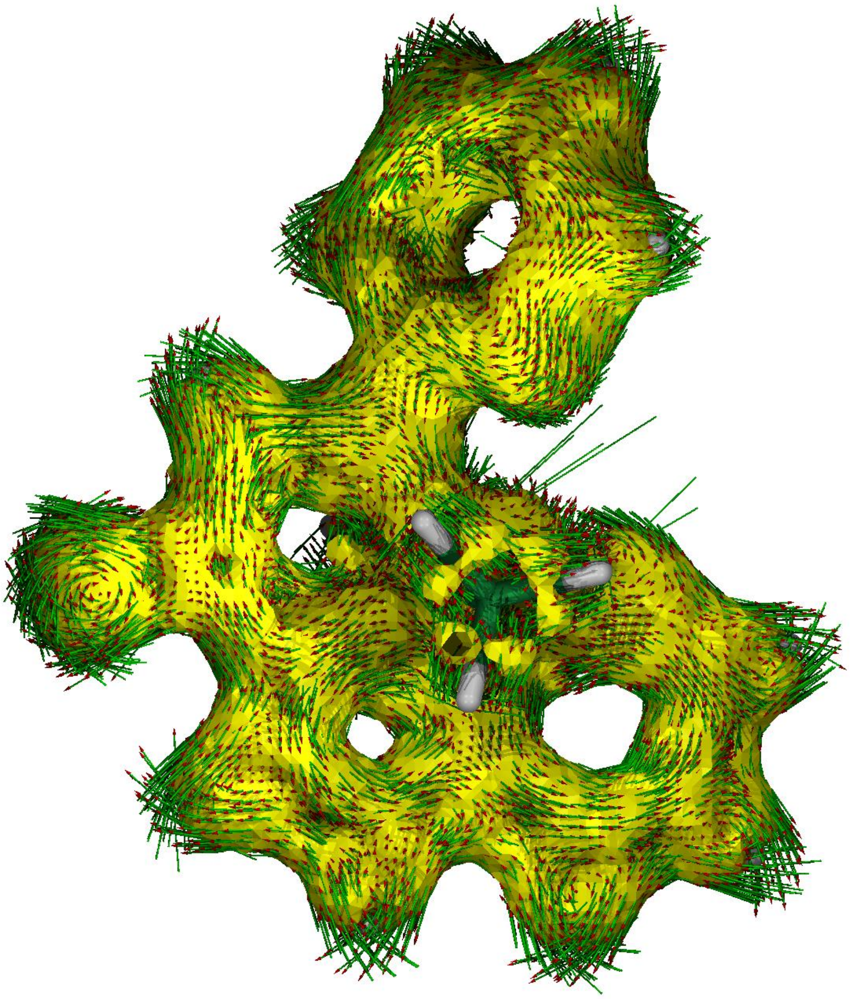

Supplementary Figure 12 | ACID plot of the model complex **3′** with an isosurface value of 0.025. The magnetic field vector is orthogonal to the ring plane and points upward (clockwise currents are diatropic).

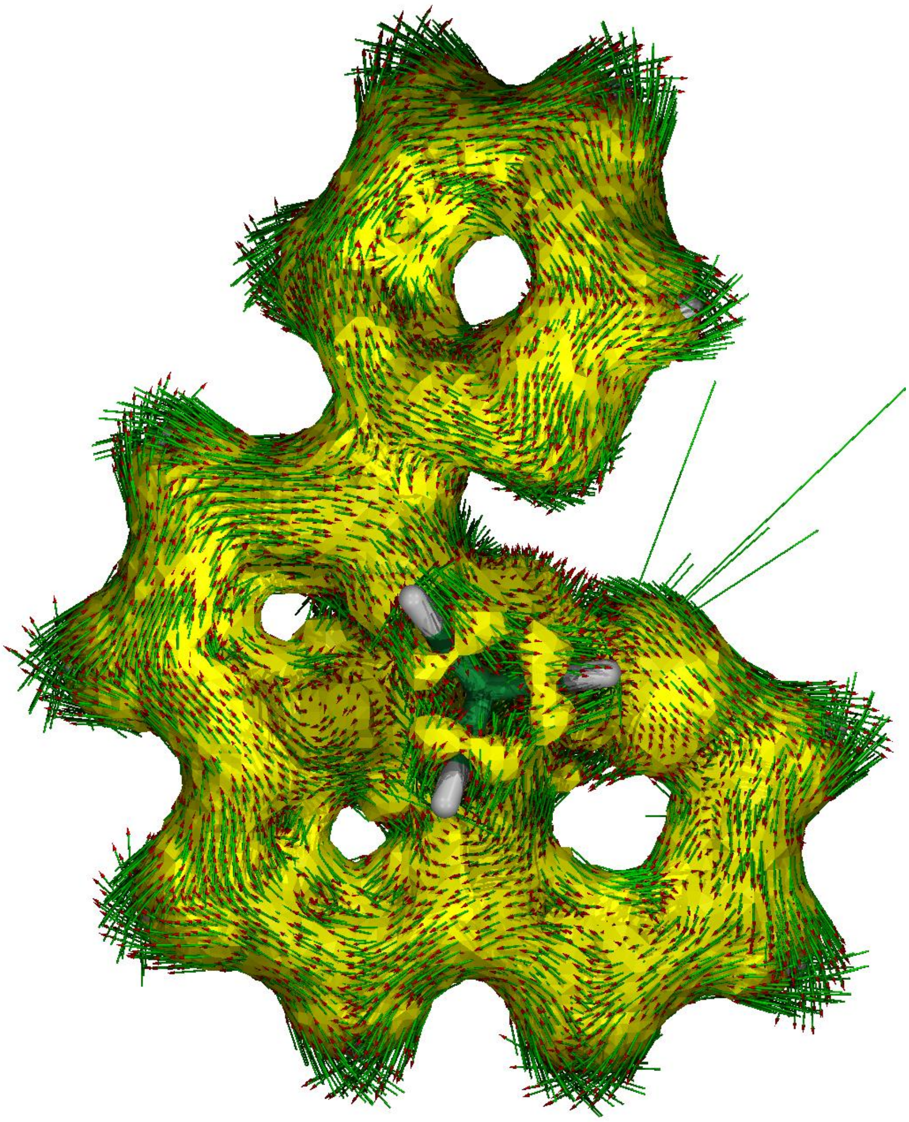

Supplementary Figure 13 | ACID plot of the model complex **4′** with an isosurface value of 0.025. The magnetic field vector is orthogonal to the ring plane and points upward (clockwise currents are diatropic).

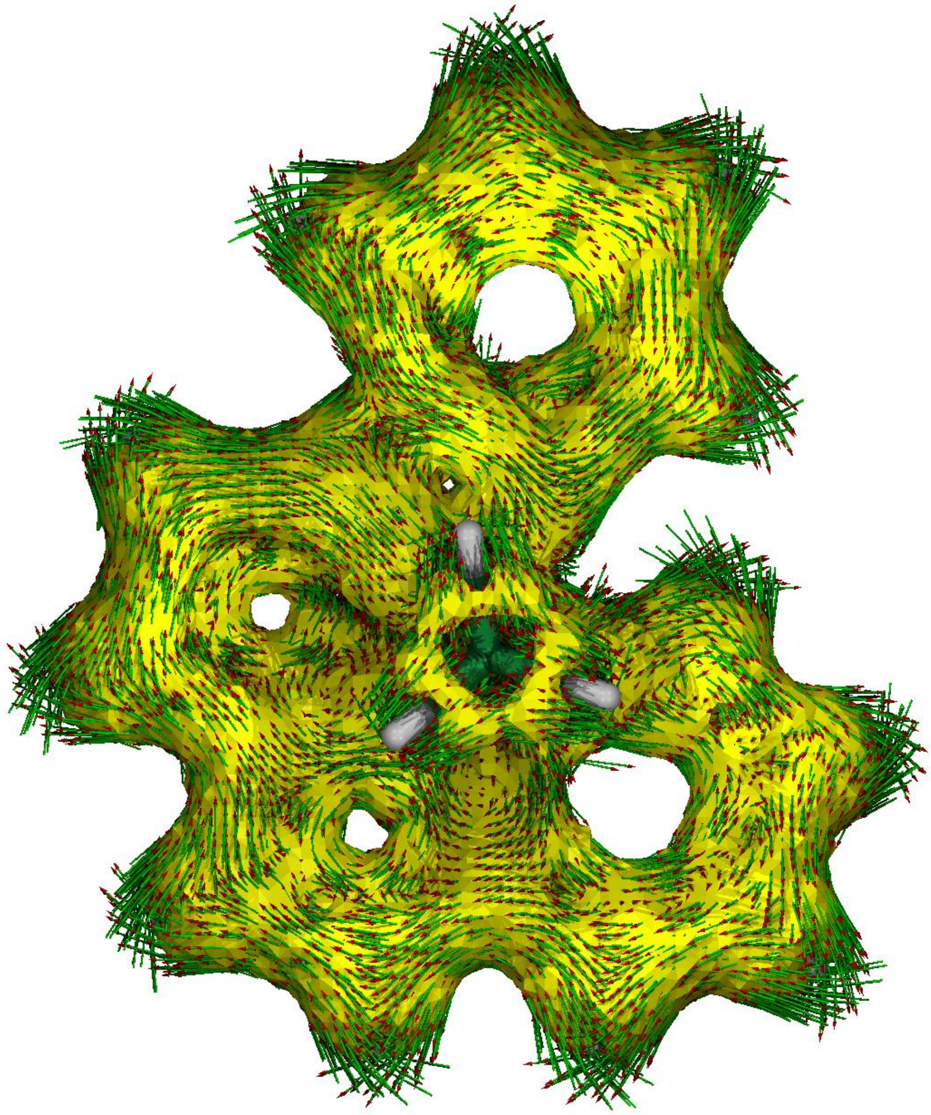

Supplementary Figure 14 | ACID plot of the model complex **5′** with an isosurface value of 0.025. The magnetic field vector is orthogonal to the ring plane and points upward (clockwise currents are diatropic).

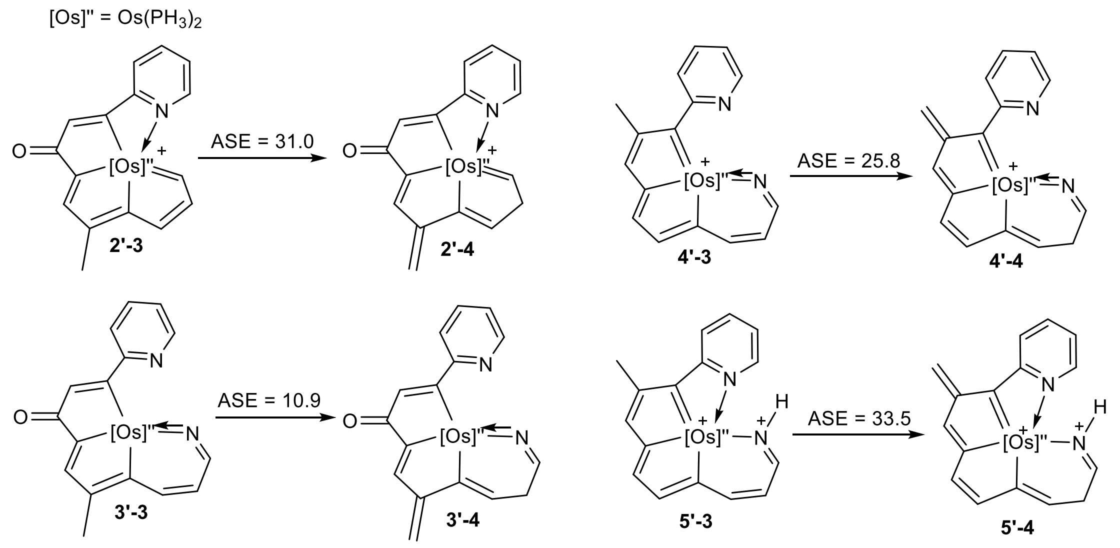

Supplementary Figure 15 | ASE evaluations of the model complexes **2′-5′**. The energies include the zero-point energy corrections (given in kcal mol^{-1}).

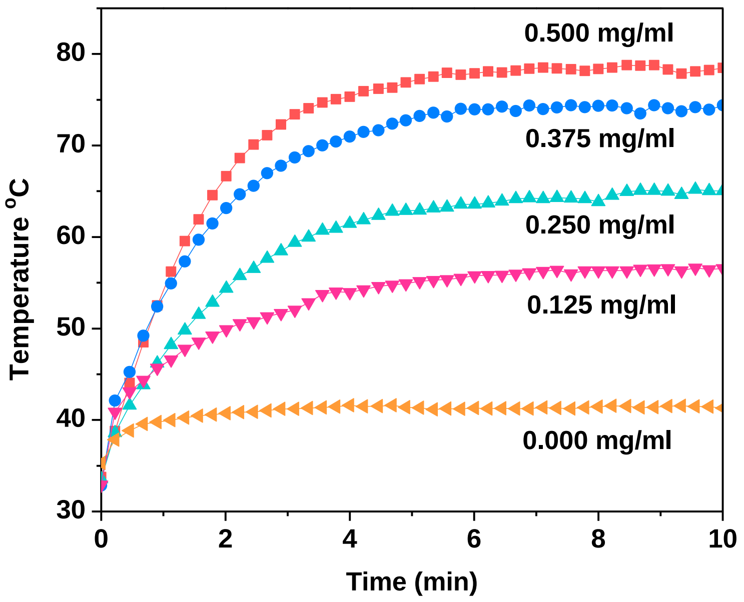

Supplementary Figure 16 | Photothermal effect of 10% Ethanol-H_2O solutions of complex **4** at 0.500/0.375/0.250/0.125 mg/ml and of the pure solvent alone upon laser irradiation (λ = 808 nm, 1.0 W cm^{-2}).

HRMS and NMR Spectra

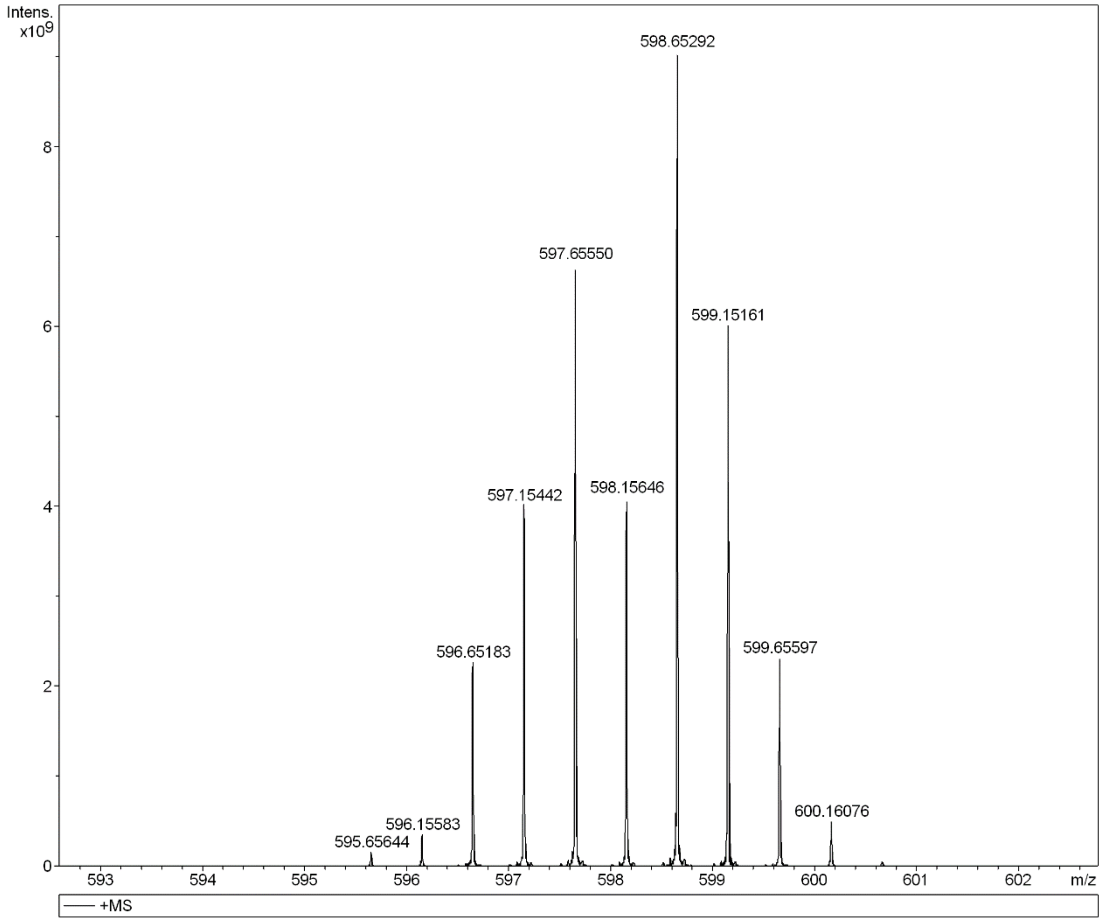

Supplementary Figure 17 | Positive-ion ESI-MS spectrum of [**2**]$^{2+}$ [$C_{69}H_{54}NOOsP_3$]$^{2+}$ measured in methanol.

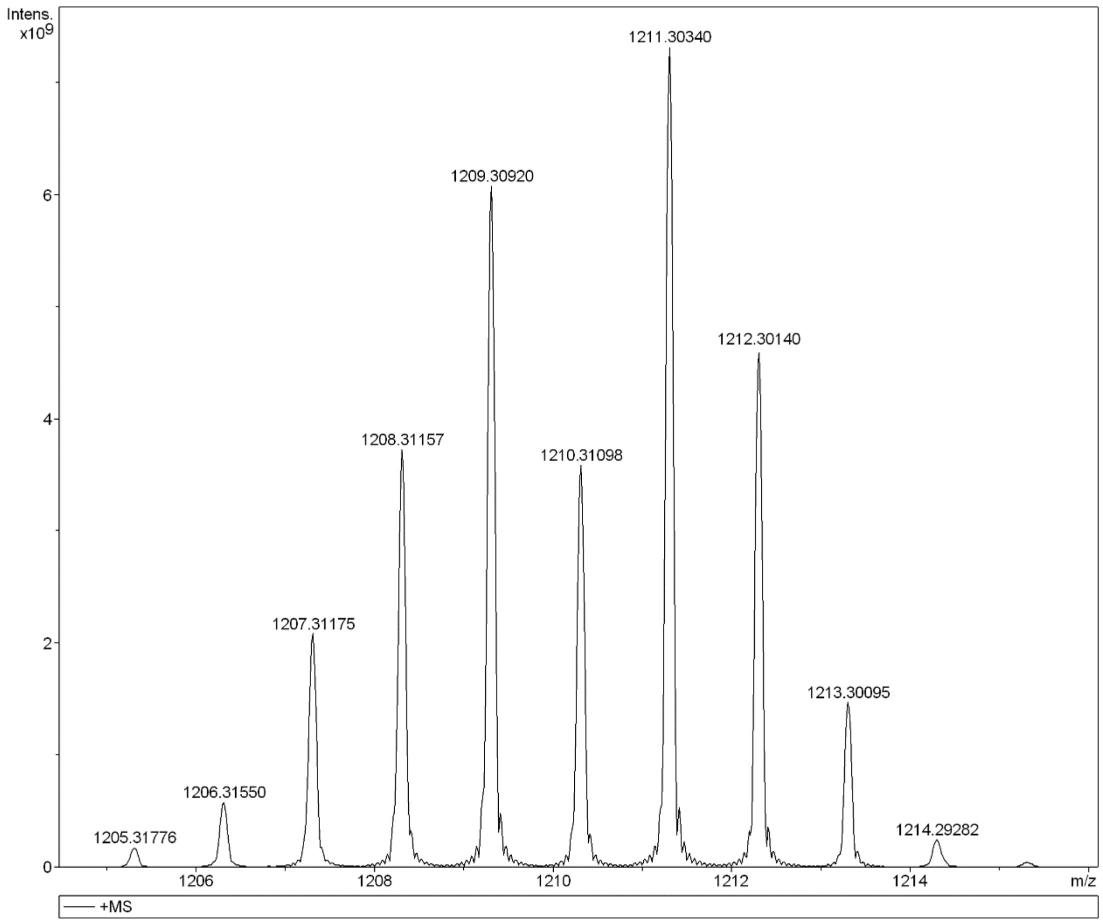

Supplementary Figure 18 | Positive-ion ESI-MS spectrum of [**3**]$^+$ $[C_{69}H_{54}N_2OOsP_3]^+$measured in methanol.

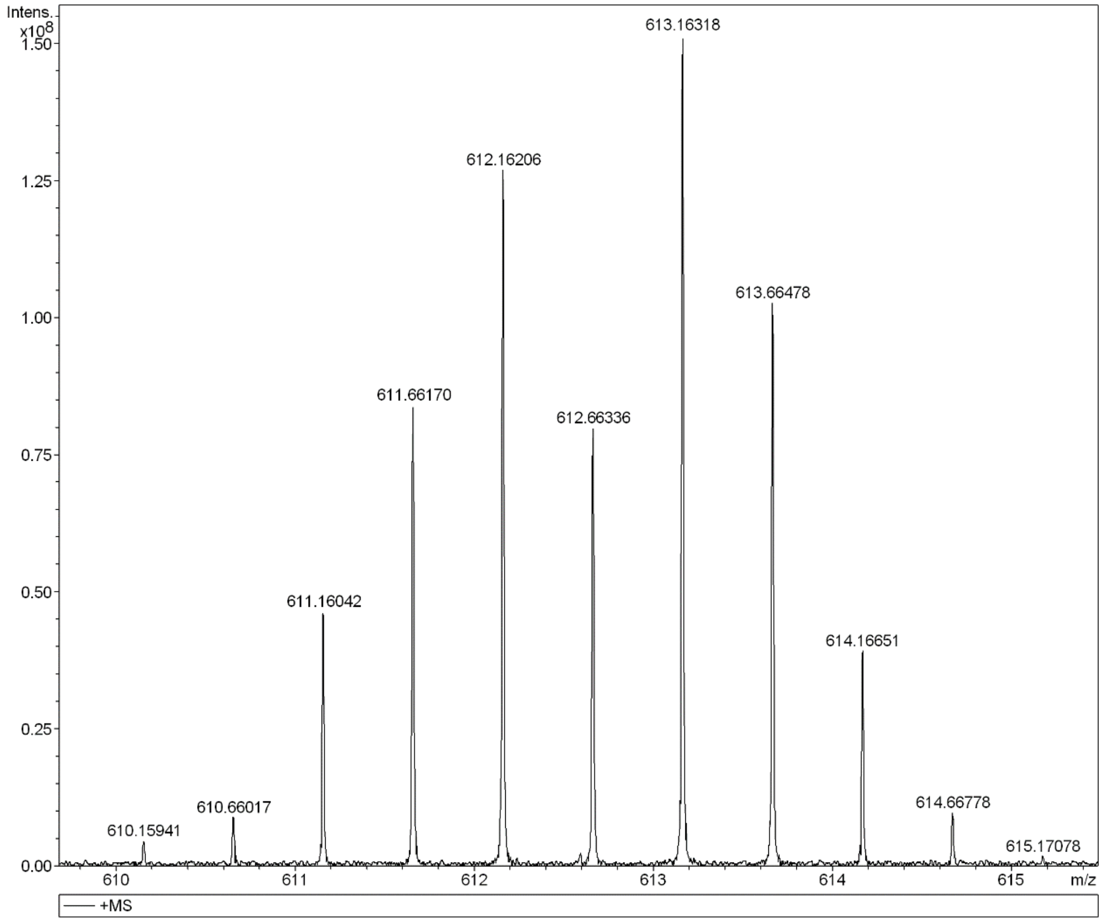

Supplementary Figure 19 | Positive-ion ESI-MS spectrum of [**4**]$^{2+}$[$C_{70}H_{57}N_2OOsP_3$]$^{2+}$ measured in methanol.

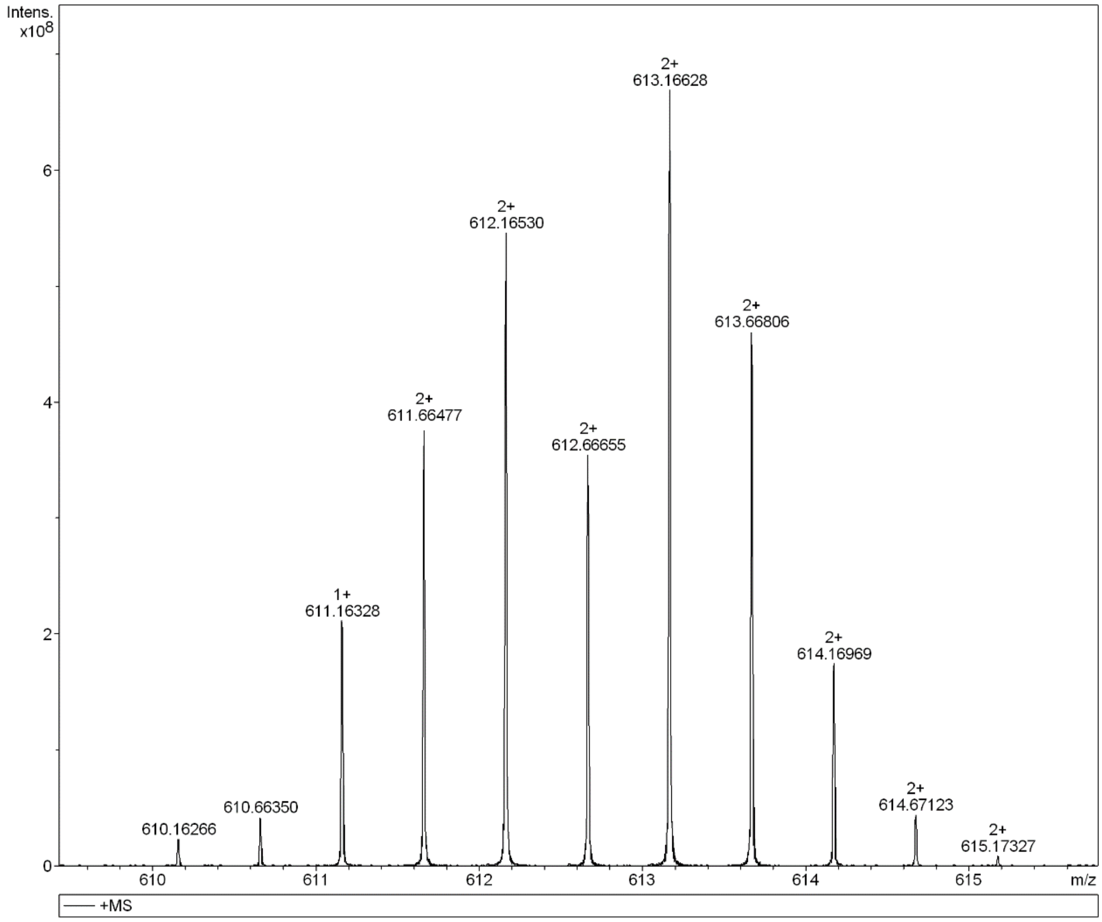

Supplementary Figure 20 | Positive-ion ESI-MS spectrum of [$\mathbf{5}^{3+}$-H^+]$^{2+}$ [$C_{70}H_{58}N_2OOsP_3^{3+}$ - H^+]$^{2+}$ measured in methanol.

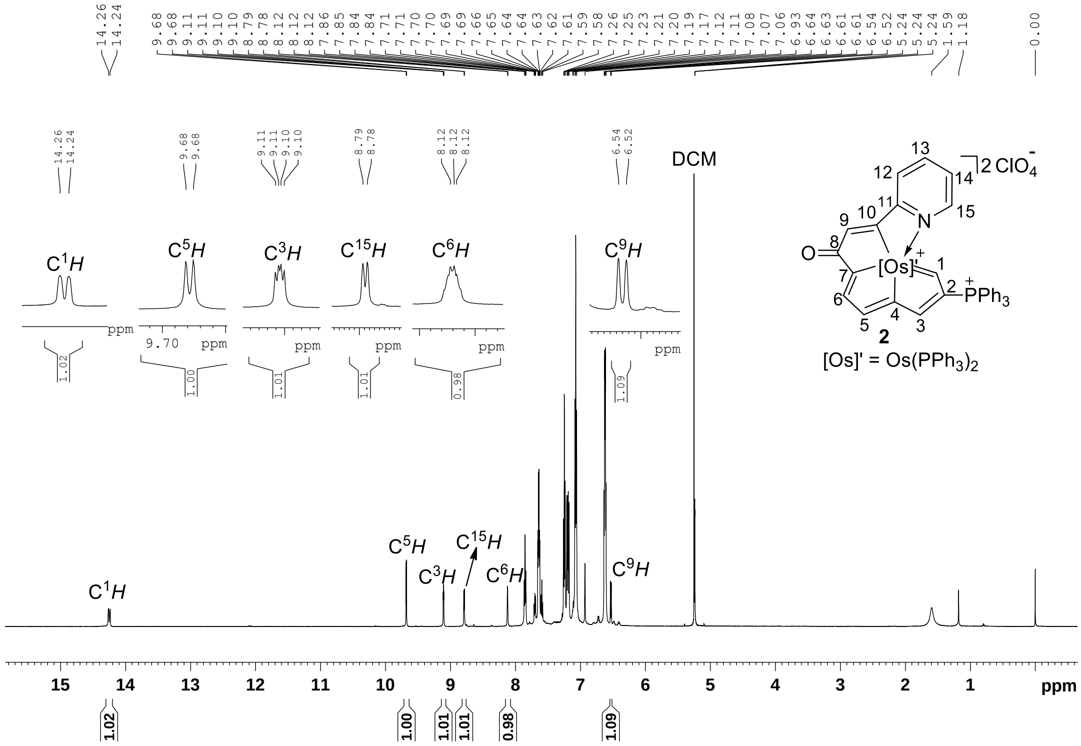

Supplementary Figure 21 | The ^{1}H NMR (600.1 MHz, CD_2Cl_2) spectrum for complex **2**.

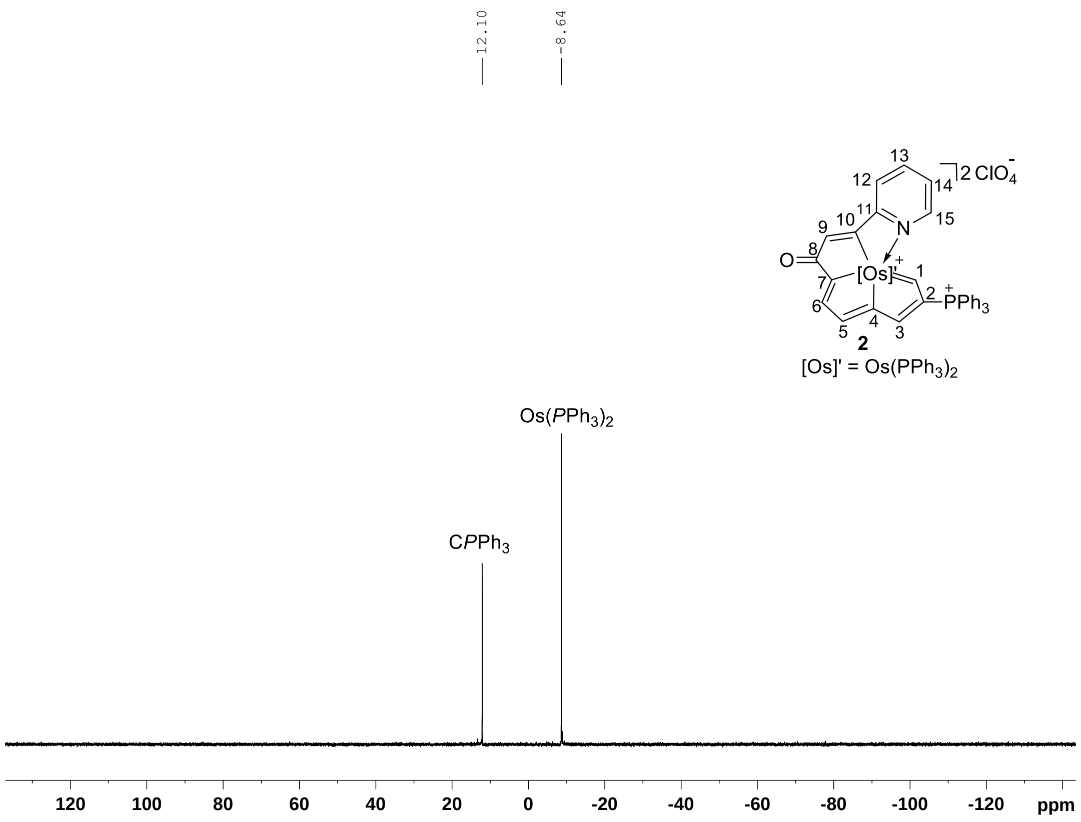

Supplementary Figure 22 | The $^{31}P\{^{1}H\}$ NMR (242.9 MHz, CD_2Cl_2) spectrum for complex **2**.

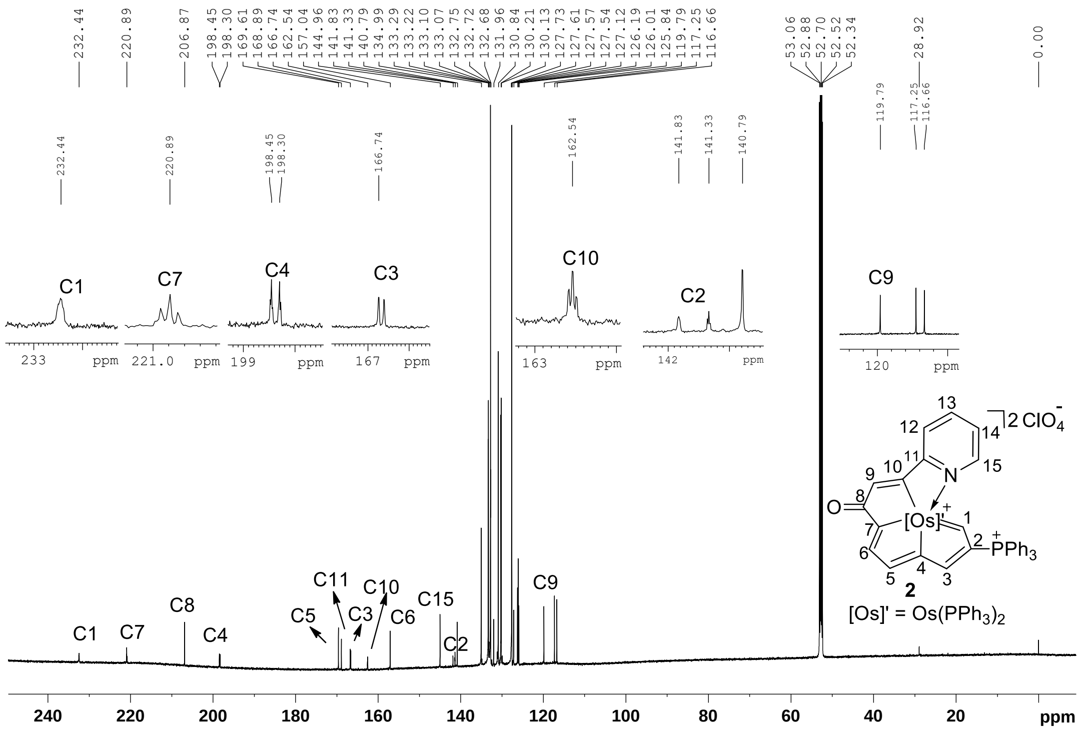

Supplementary Figure 23 | The $^{13}C\{^{1}H\}$ NMR (150.9 MHz, CD_2Cl_2) spectrum for complex **2**.

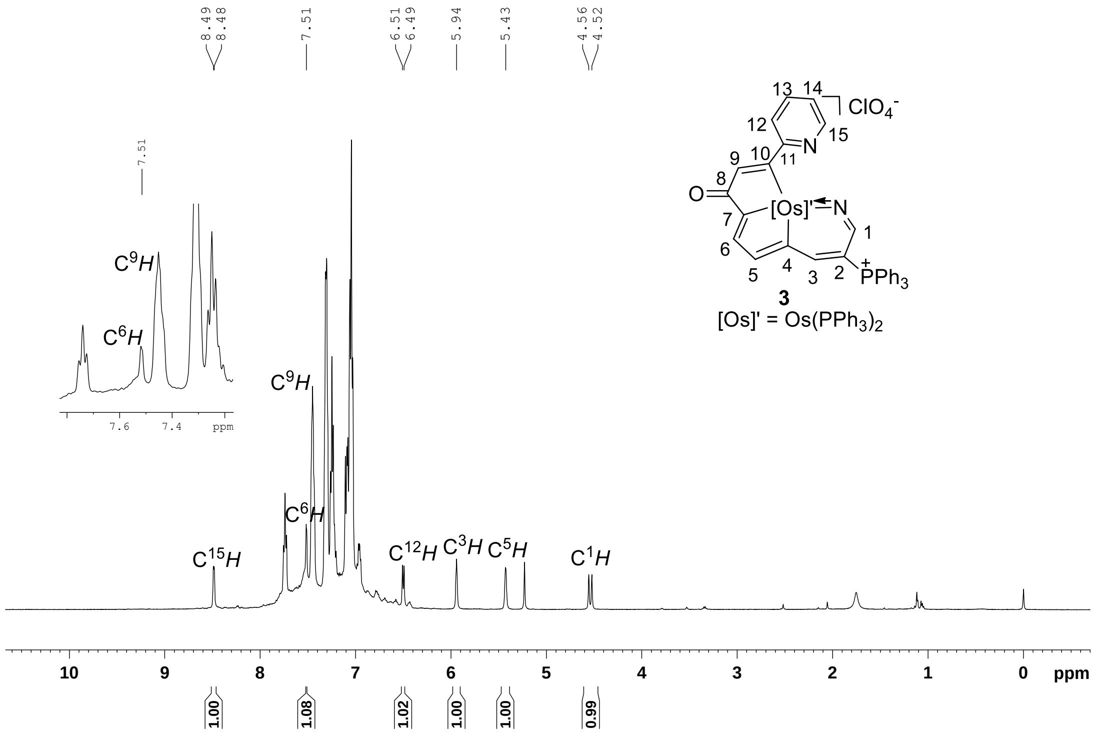

Supplementary Figure 24 | The ^{1}H NMR (500.2 MHz, CD_2Cl_2) spectrum for complex **3**.

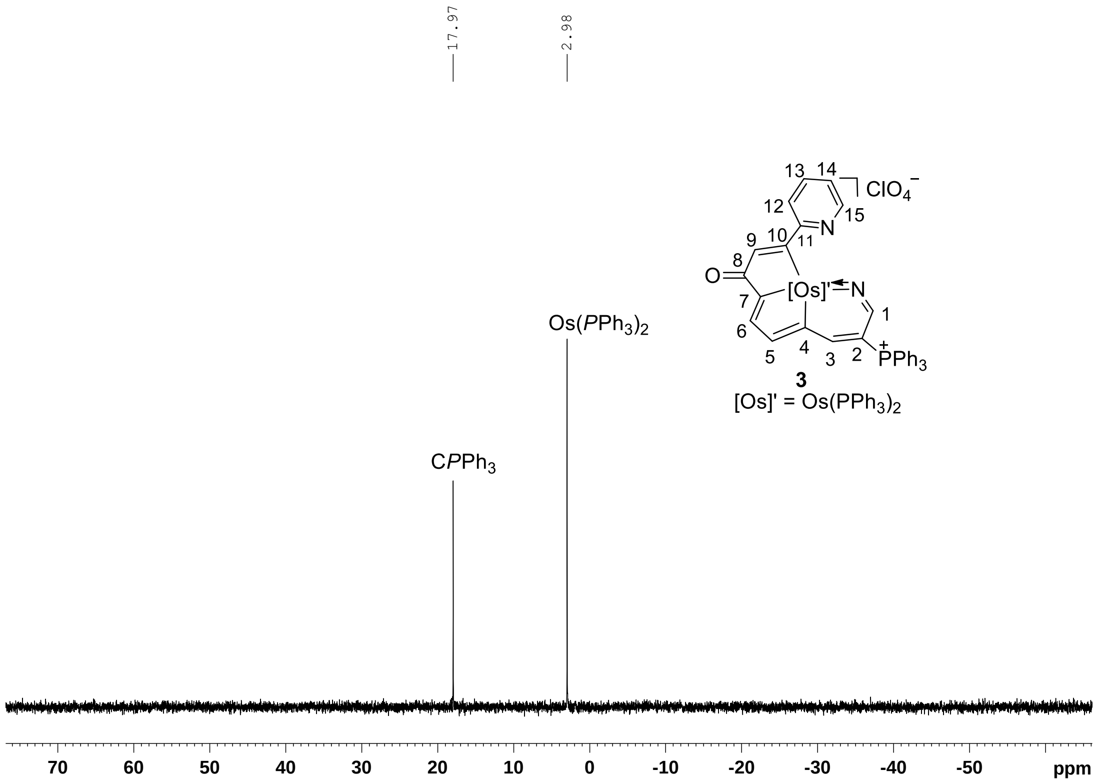

Supplementary Figure 25 | The $^{31}P\{^1H\}$ NMR (202.5 MHz, CD_2Cl_2) spectrum for complex **3**.

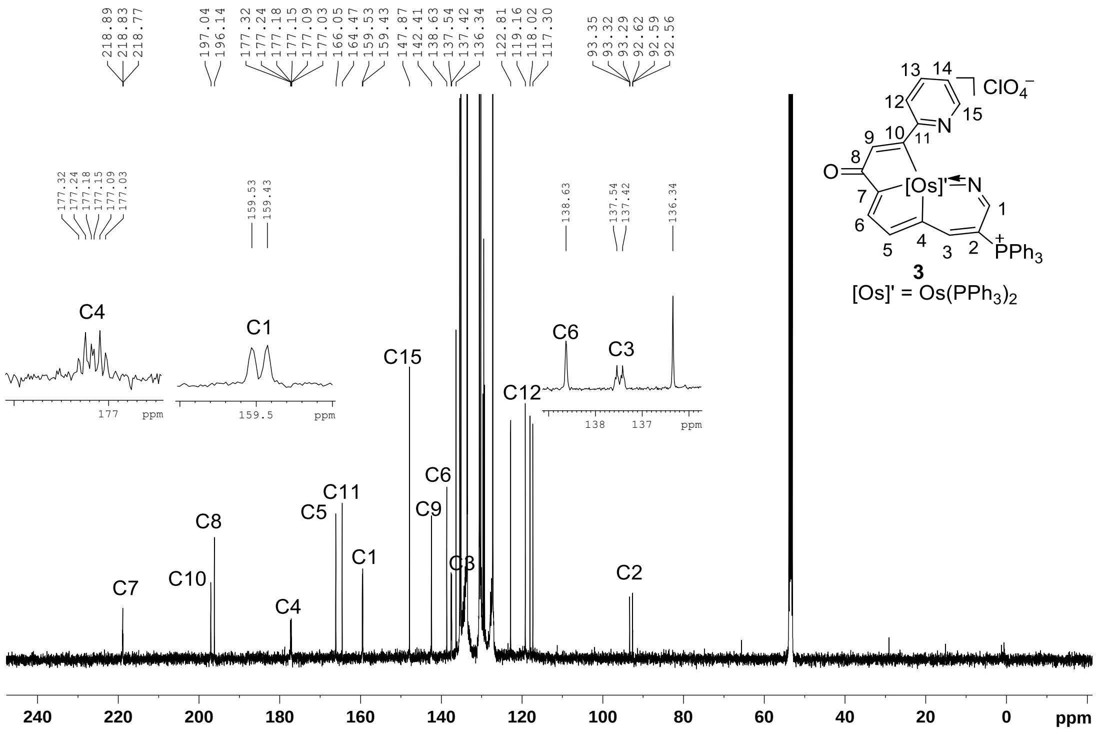

Supplementary Figure 26 | The $^{13}C\{^{1}H\}$ NMR (125.8 MHz, CD_2Cl_2) spectrum for complex **3**.

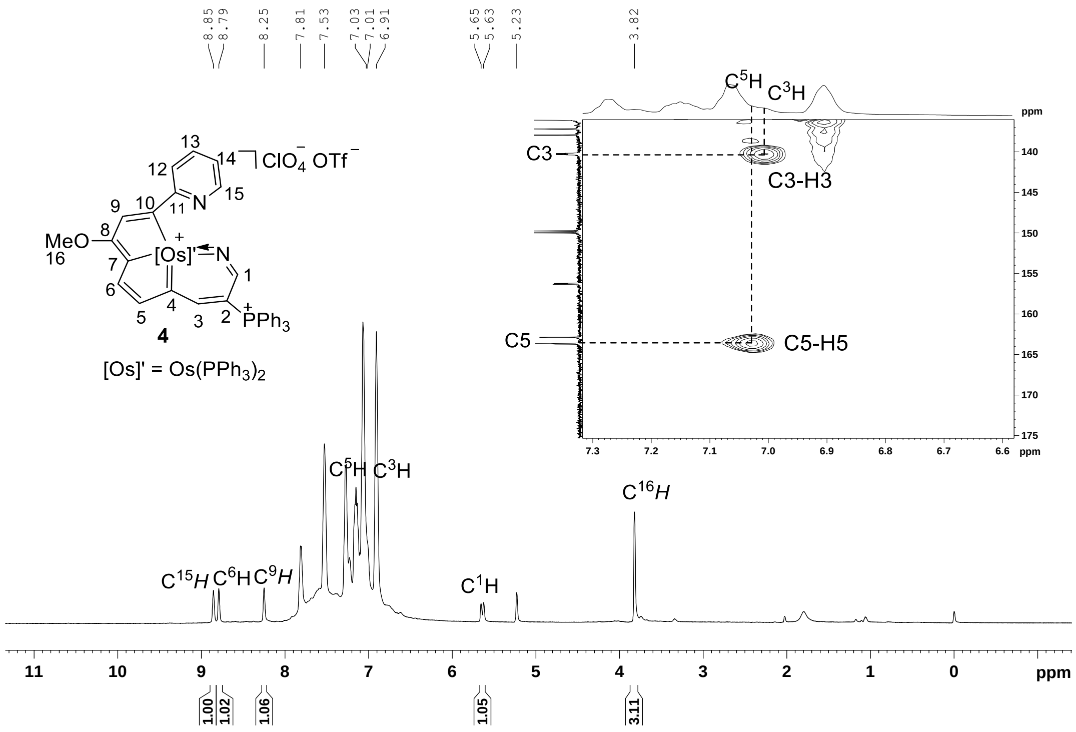

Supplementary Figure 27 | The ^{1}H NMR (500.2 MHz, CD_2Cl_2) spectrum (Inset: partial ^{1}H-^{13}C HSQC spectrum) for complex **4**.

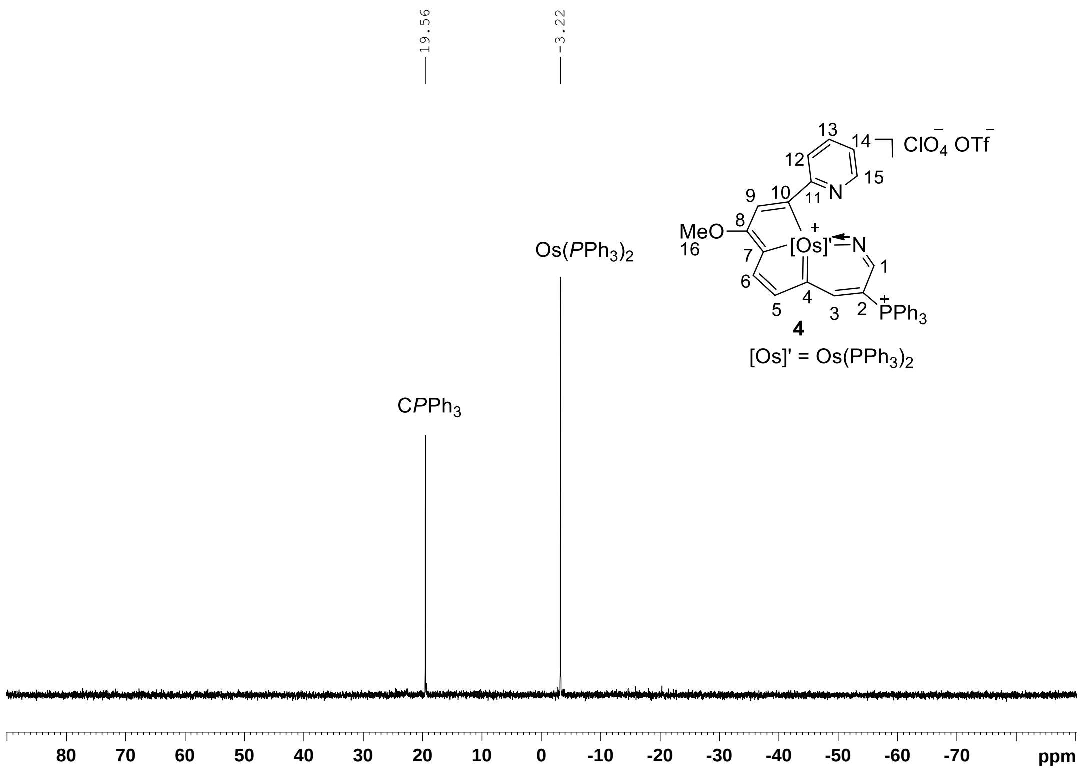

Supplementary Figure 28 | The $^{31}P\{^{1}H\}$ NMR (202.5 MHz, CD_2Cl_2) spectrum for complex **4**.

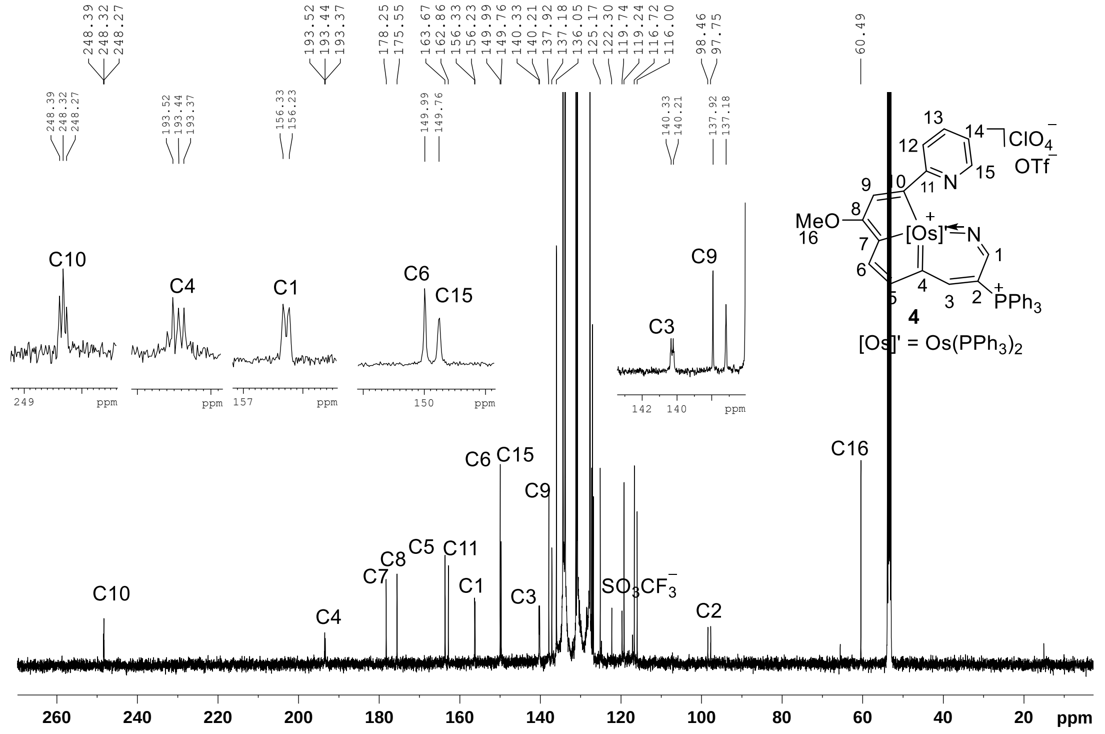

Supplementary Figure 29 | The $^{13}C\{^{1}H\}$ NMR (125.8 MHz, CD_2Cl_2) spectrum for complex **4**.

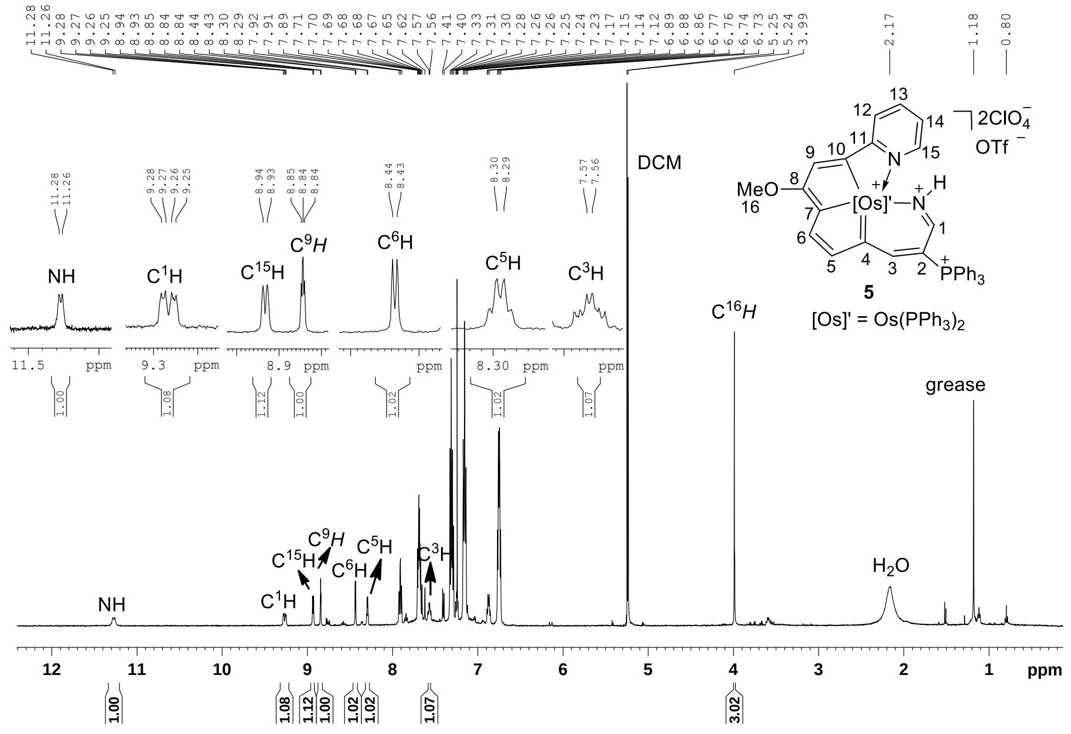

Supplementary Figure 30 | The ^1H NMR (500.2 MHz, CD_2Cl_2) spectrum for complex **5**.

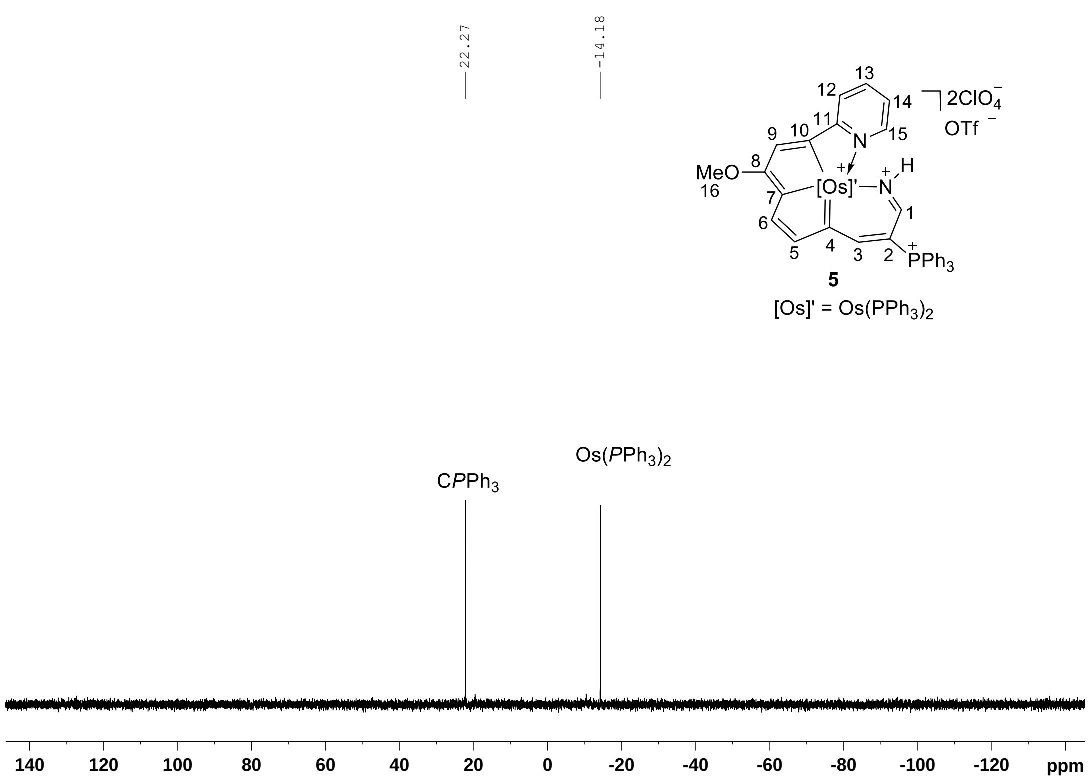

Supplementary Figure 31 | The $^{31}P\{^{1}H\}$ NMR (202.5 MHz, CD_2Cl_2) spectrum for complex **5**.

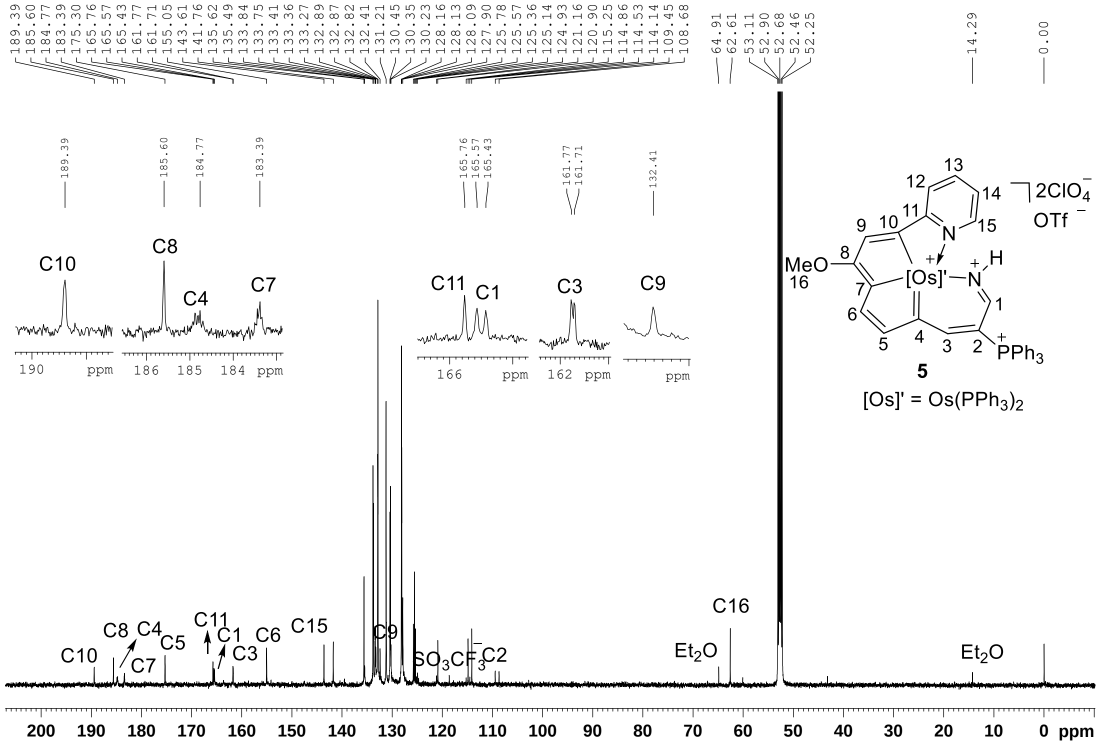

Supplementary Figure 32 | The $^{13}C\{^1H\}$ NMR (125.8 MHz, CD_2Cl_2) spectrum for complex **5**.
