# Supplementary material for: Successive modification of polydentate complexes gives access to planar carbon- and nitrogen-based ligands
Source: Nat Commun. 2019 Apr 2;10:1488. doi: 10.1038/s41467-019-09367-8 (PMC6445293; doi:10.1038/s41467-019-09367-8)
Supplement: Supplementary file 2 — Description of Additional Supplementary Files [file 41467_2019_9367_MOESM2_ESM.docx]

Description of Additional Supplementary Files

**Supplementary Data:** XYZ coordinates of the optimized structures in the DFT studies
